# Characterizing the binding and function of TARP γ8-selective AMPA receptor modulators

**DOI:** 10.1074/jbc.RA120.014135

**Published:** 2020-08-03

**Authors:** Jan-Niklas Dohrke, Jake F. Watson, Kristian Birchall, Ingo H. Greger

**Affiliations:** 1 Neurobiology Division, Medical Research Council Laboratory of Molecular Biology, Cambridge, United Kingdom; 2 LifeArc, Open Innovation Campus, Stevenage, United Kingdom

**Keywords:** synapse, α-amino-3-hydroxy-5-methyl-4-isoxazolepropionic acid receptor (AMPA receptor, AMPAR), glutamate receptor, ionotropic glutamate receptor, electrophysiology, AMPA receptor, MD simulations

## Abstract

α-amino-3-hydroxy-5-methyl-4-isoxazolepropionic acid(AMPA)-type glutamate receptors (AMPARs) are the predominant excitatory neurotransmitter receptors in the brain, where they mediate synaptic transmission and plasticity. Excessive AMPAR activation leads to diseases such as epilepsy. AMPAR properties are modulated by auxiliary proteins and foremost by the transmembrane AMPAR regulatory proteins (TARPs). These distribute in unique expression patterns across the brain, rendering AMPAR/TARP complexes promising targets for region-specific therapeutic intervention. TARP γ8 is predominantly expressed in the forebrain and is enriched in the hippocampus, a region associated with temporal lobe epilepsy. Recent high-throughput medicinal chemistry screens have identified multiple promising compounds that selectively target AMPARs associated with γ8 and hold promise for epilepsy treatment. However, how these modulators target the receptor complex is currently unknown. Here, we use a combination of ligand docking, molecular dynamics simulations, and electrophysiology to address this question. We identify a conserved oxindole isostere, shared between three structurally diverse modulators (LY-3130481, JNJ-55511118, and JNJ-61432059) as the major module engaging γ8 by an H-bond to Asn-172 (γ8). The remaining variable region of each molecule likely targets the receptor complex in ligand-selective modes. Functional data reveal parallels in the underlying modulatory action of two prominent compounds. This work will aid development of refined AMPAR epilepsy therapeutics and facilitate to uncover the mechanisms by which TARPs modulate the receptor.

α-amino-3-hydroxy-5-methyl-4-isoxazolepropionic acid (AMPA) receptors (AMPARs) are glutamate-gated cation channels that mediate fast excitatory neurotransmission throughout the central nervous system (1). The regulation of AMPARs is central to synaptic plasticity, which underlies higher cognitive brain functions such as learning and memory (2). Malfunction of these receptors is associated with a variety of neurological and psychiatric disorders, rendering them a strategic drug target (3). AMPAR-targeting therapeutics that have advanced into clinical trials are either positive allosteric modulators that improve cognition (4–6) or negative allosteric modulators (NAMs) that have been trialed in epilepsy treatment (7). Nevertheless, because both modulator types target sequence-conserved receptor segments, the ligand-binding domain in the case of positive allosteric modulators (5) and the channel gate region for NAMs (8, 9) (see Fig. 1*A*), they will act broadly on AMPARs across the brain, causing un-wanted side effects. More recently, progress has been made to achieve brain region specificity by selectively targeting auxiliary subunits that associate with the AMPAR core subunits (10–14).

**Figure 1.**
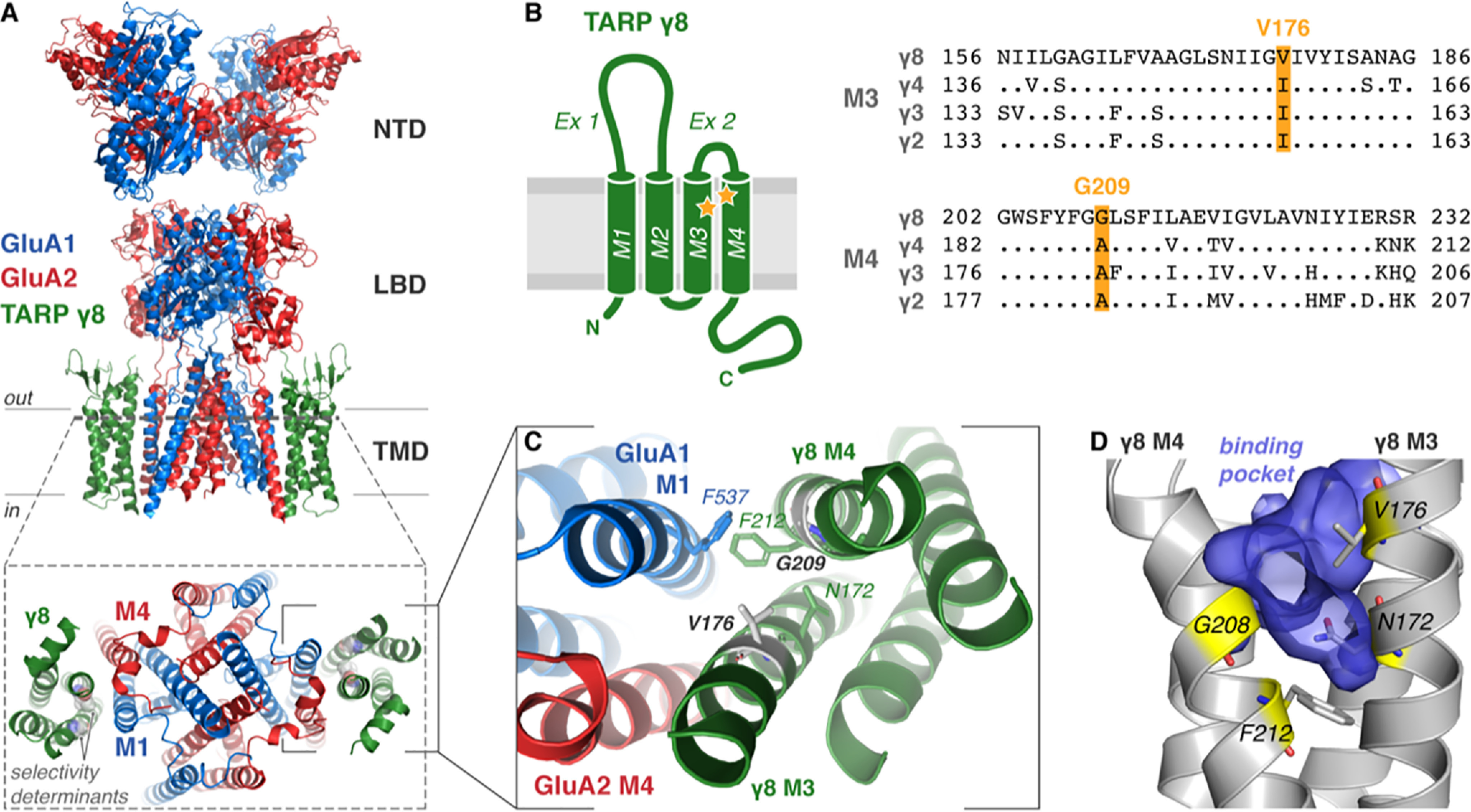
**Architecture and proposed ligand-binding pocket of TARP γ8.** *A*, cryo-EM structure of heteromeric GluA1/2 AMPAR (*blue*/*red*, respectively) in complex with TARP γ8 (*green*; PDB 6QKZ) demonstrating the domain architecture (NTD, N-terminal domain; LBD, ligand-binding domain; TMD, transmembrane domain). A top view onto the pore region (*bottom,* from *dotted slice*) demonstrates γ8 association with M1 and M4 of the AMPAR. γ8 ligand selectivity-determining residues Gly-209 and Val-176 are shown (*gray spheres*). *B*, TARP topology schematic shows the four transmembrane helices (TM1–4) with two extracellular loop elements (*Ex1* and *Ex2*) and both N and C termini located intracellularly. Sequence alignment of type I TARPs (*Rattus norvegicus*) with divergent residues only shown for TARPS γ2–4 (*right*) highlights the residues determining γ8 selectivity of ligands (*yellow*, Val-176 and Gly-209), also marked on the topology map (*yellow stars*). *C*, expanded view of the AMPAR-γ8 interaction interface viewed from above. Critical interface residues are depicted as *sticks*, and selectivity-determining residues are *gray*. *D*, the important ligand-binding residues (*yellow sticks*, see Maher *et al*. 2016 (10)) line the surface of a binding pocket (*blue volume*) of an all-atom model of γ8 that forms in MD simulations between TM3 and TM4 of γ8 (*gray*).

AMPARs are tetramers that assemble from four core subunits, GluA1–4, in various combinations (15). Akin to voltage-gated ion channels, AMPARs associate with a multitude of auxiliary subunits (16), mostly transmembrane proteins, that facilitate receptor trafficking and modulate gating kinetics, ion flux, and receptor pharmacology (17, 18). The first identified and best characterized are the transmembrane AMPAR regulatory proteins (TARPs) (19, 20), tetraspanin-like proteins that are classified into three subgroups based on sequence conservation and modulatory action: type 1a (γ2 and γ3), type 1b (γ4 and γ8), and type 2 (γ5 and γ7) (21, 22). TARPs generally slow gating kinetics, prolonging receptor activation, and are expressed in distinct, partially overlapping patterns in the brain. The first identified TARP, γ2 (or stargazin), is predominantly expressed in the cerebellum; accordingly, γ2 mouse mutants show severe deficits in motor coordination (23). TARP γ8 predominates in the forebrain and is the major TARP in the hippocampus (21, 24), where AMPARs are predominantly associated with γ8 and another auxiliary subunit, cornichon-homologue 2 (25–27). Cryo-EM structures revealed how these proteins associate with the receptor (Fig. 1*A*), docking to the outer transmembrane AMPAR helices, M1 and M4 (Fig. 1, *A***–***C*) (28, 29), a finding that has been confirmed through functional studies (30).

High-throughput screening and chemical optimization led to the discovery of chemically diverse NAMs that selectively target AMPAR-γ8 complexes but were ineffective on other TARPs (10, 11). Sequence analysis and mutagenesis identified a potential binding site for these drugs between TARP γ8 (transmembrane helices M3_T_ and M4_T_) and the AMPAR (helices M1_A_ and M4_A_). TARP-selectivity is conferred by two residues unique to γ8, Val-176 in M3_T_ and Gly-209 in M4_T_ (rat sequence) (Fig. 1, *B*–*D*), that are replaced in the other type 1 TARPs (γ2–4) by the bulkier isoleucine and alanine (Fig. 1*B*), which likely block ligand access. These modulators, together with more potent new derivatives (13), are promising candidates for treating disorders characterized by enhanced excitatory neurotransmission such as epilepsy (31, 32) and pain therapy (33).

The cryo-EM structure of the GluA1/2 AMPAR heteromer associated with γ8 (34) permits a first characterization of the binding modes of these modulators at the molecular level. Toward this aim we combined rigid and induced-fit docking with all-atom molecular dynamics (MD) simulations and electrophysiology to investigate how these ligands target the AMPAR-γ8 complex. We identify a conserved binding pose shared between three chemically diverse modulators and describe a major functional component contributing to the negative modulation of receptor gating. Our data shed new light on the function of AMPAR-γ8 modulators and will permit structure-based refinement of improved derivatives.

## Results

### Structural comparisons of three distinct γ8 modulators

**Figure 2.**
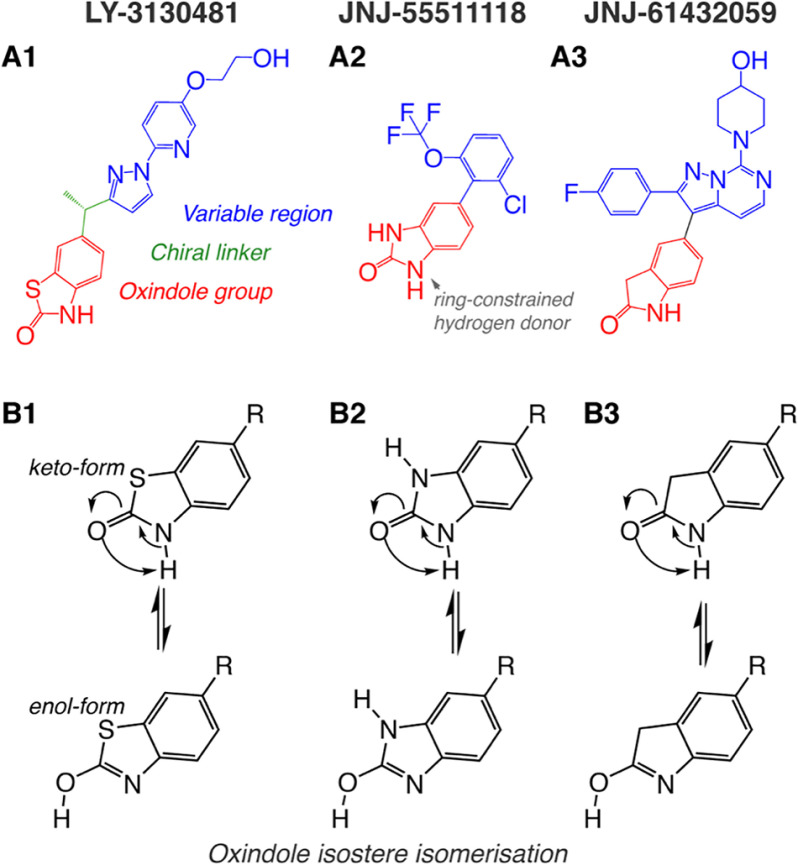
**TARP γ8 selective negative allosteric modulators.** *A*, the atomic arrangement of modulatory compounds, highlighting the oxindole isostere group common to all compounds (*red*; LY-281, benzothiazolone; JNJ-118, benzimidazolone; JNJ-059, oxindole) and a more structurally diverse region (*blue*, *variable region*). LY-3130481 (*A1*) has a chiral linker (*green*) between these regions. LY-481 is the more active S-enantiomer. *B*, all compounds may form alternative tautomers with a hydroxyl form of the oxindole group, confirmed by NMR spectroscopy for LY-3130481 (*B1*), but not detected for JNJ-61432059 (*B3*), and indistinguishable for JNJ-55511118 (*B2*). The rest (*R*) in the *bottom row* corresponds to the variable and linker regions of the ligands depicted in the *top row*.

The AMPAR-γ8 modulators investigated in this study, LY-3130481, JNJ-55511118, and JNJ-61432059 (herein referred to as LY-481, JNJ-118, and JNJ-059), are depicted in Fig. 2*A*. LY-481 and JNJ-118 are the best characterized, blunting some of the positive modulatory action conferred by TARPs (10, 11), whereas JNJ-059 is a more recently reported compound, with substantially greater potency and of promise in rodent seizure models (13). All three ligands share an oxindole isostere moiety (Fig. 2*A*, *red substructure*), which is critical for their potency, as determined by lead optimization campaigns (12, 13, 35) (see also Fig. S1). Specifically, the presence of a ring-constrained hydrogen donor in this substructure was shown to be crucial for activity (27) (Fig. 2*A*). The variable region (Fig. 2*A*, *blue*) differs substantially between ligands, and contrary to the oxindole isostere, alteration of this region never causes compound inactivation, suggesting that it is not a critical region for protein interaction (12, 13, 35) (Fig. S1). Interestingly, chiral activity of LY-481, mediated by the carbon linker between oxindole and variable regions (Fig. 2*A1*, *green*), had a substantial impact on binding, with the (S)-enantiomer being two orders of magnitude more potent than the (R)-enantiomer (pIC_50_ = 7.2 *versus* 5.1) (12). Because radiolabeled LY-481 derivatives can associate with free γ8 in the absence of the AMPAR (36), we hypothesized that the oxindole isostere could directly engage with γ8 to mediate the compounds' effects.

We considered the possibility that the oxindole isosteres also exist as enol tautomers (in addition to the published keto isomers), which is apparent from ^1^H-NMR spectra of related structures in the Spectral Database for Organic Compounds (SDBS). ^1^H NMR spectra permit the assignment of hydrogen atoms to specific groups, because the spectra are influenced by surrounding atoms within the molecule (37). Whereas the oxindole group of JNJ-059 favors the keto tautomer (SBDS 13584) (Fig. 2*B*, *top row*), the benzimidazolone of JNJ-118 could exist in either the keto or the enol form (SBDS 13287; Fig. 2*B*). Interestingly, for the LY-481 benzothiazolone moiety, the enol form appeared dominant (SBDS 17222). Based on these observations, both tautomers were used for ligand docking.

### Rigid docking suggests oxindole group engagement with γ8 for all modulators

To enable ligand docking, we first generated an all-atom model based on the GluA1/2 TARP γ8 cryo-EM structure (PDB 6QKC) (34) in MODELLER (38). The best model was chosen following two criteria: its discrete optimized protein energy (DOPE) score (39) and its root mean square deviation (RMSD), providing a low-energy model with least deviation from the input structure (PDB 6QKC). Based on these criteria, model 8 ranked highest of ten (see Table 1; “Experimental procedures”). The main goal was to select a model that also includes the intra- and extracellular AMPAR loops with a realistic conformation, while preserving the transmembrane sector. Of note, the local resolution of the cryo-EM map in the transmembrane region, including the binding site, was < 4 Å, providing a realistic starting structure (34).

**Table 1:** **Model quality assessment** All-atom models of 6QKC were generated using MODELLER (38). The RMSD to the cryo-EM structure 6QKC and the DOPE score of MODELLER were computed as presented above (39).

Model no.	RMSD	DOPE score
1	1.14	−227166
2	1.05	−226840
3	1.01	−227057
4	1.09	−226705
5	1.09	−225611
6	1.13	−227439
7	1.15	−226287
8	1.05	−229642
9	1.11	−227128
10	1.07	−226868

**Figure 3.**
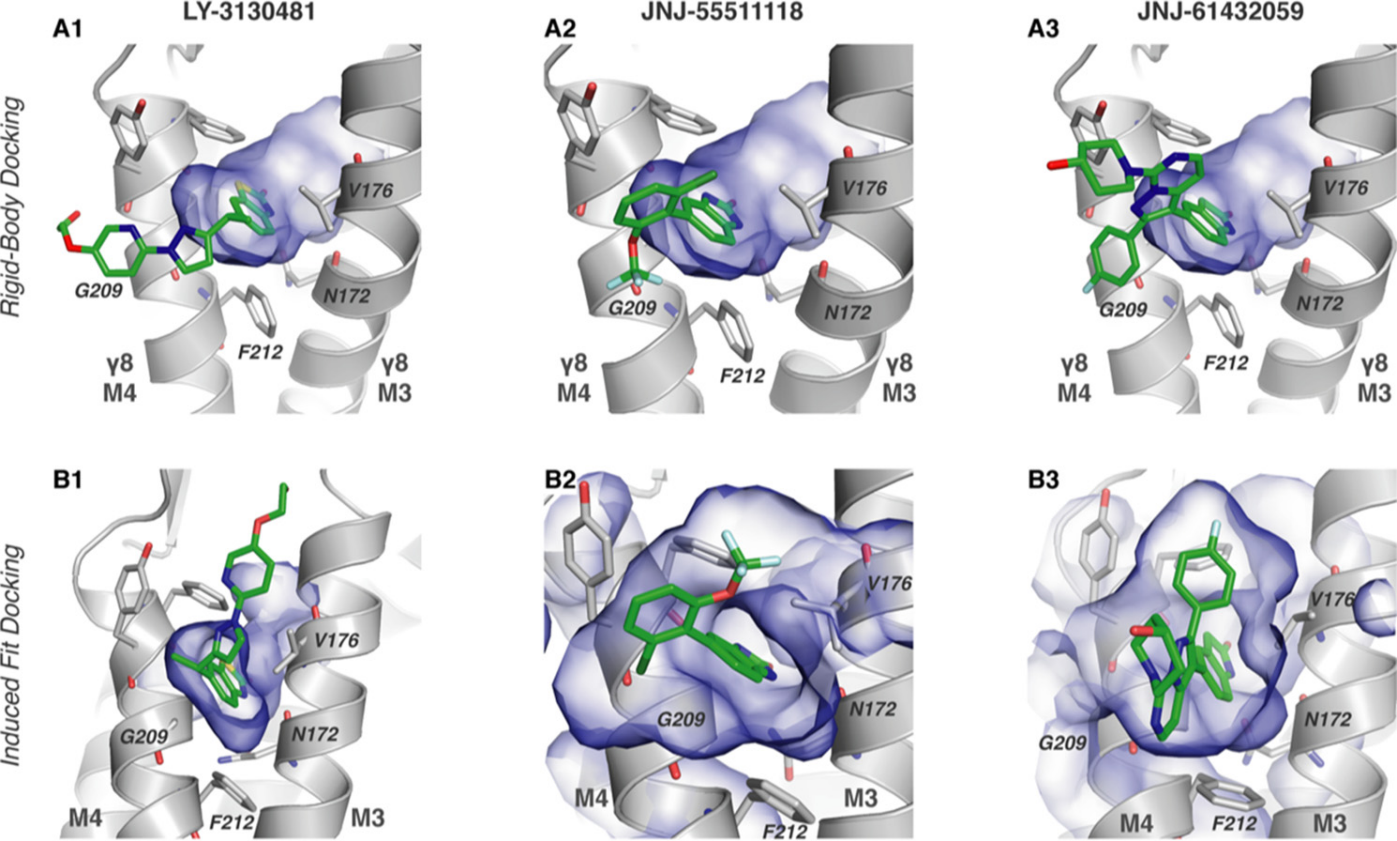
**Predicted docking poses for modulatory compounds.** *A*, poses for compound docking in a snapshot of rigid-body docking with the AMPAR-γ8 complex (30.18-ns snapshot from 50-ns simulation; γ8 depicted in *gray*, binding residues as labeled *sticks* surrounding *blue* volume of the binding pocket). *B*, binding poses from induced-fit docking for the three modulatory compounds in the initial structure. In all panels, the binding site is viewed from the AMPAR toward the TARP.

Mutagenesis studies highlighted two γ8 specific residues in the transmembrane sector as determinants for ligand potency: Val-176 in M3_T_ and Gly-209 in M4_T_, with additional functionality imparted by surrounding residues (Asn-172, Gly-208, and Phe-212) (10, 11) (Fig. 1, *B*–*D*). We initially modeled ligand binding through a rigid docking approach, in which all protein atoms are fixed and ligands are screened in a variety of conformations (40). Because the space surrounding the γ8 specificity residues is limited (Fig. 1*D*), rigid docking with AutoDock Vina failed (40). To generate a suitable substrate for docking, we used all-atom MD simulations, allowing the protein to sample other conformations close to its energetic minimum. Widening between the γ8 selective Val-176 and Gly-209 residue Cα atoms was observed, increasing the size of the ligand-binding pocket (Fig. S2). We sampled dynamics of γ8 alone (over a 200-ns trajectory) and compared this to the behavior of GluA1/2 with γ8 (50 ns), both embedded into a realistic lipid environment (Table 2) using the CHARMM force field (41). Snapshots exhibiting maximal distance between Val-176 and Gly-209 were selected for ligand docking (Fig. 3). As expected, free γ8 was more flexible than γ8 in complex with the AMPAR, but in both simulations an overall comparable dilation of the pocket relative to the EM structure was evident (Fig. S2).

**Table 2:** **Lipid bilayer composition** Cholesterol (CHOL); 3-stearoyl-2-oleoyl-d-glycero-1-phosphatidylcholine (SOPC); 3-palmitoyl-2-oleoyl-d-glycero-1-phosphatidylcholine (POPC); *N*-stearoyl-d-erythro-sphingosylphosphorylcholine (SSM); *N*-(15Z-tetracosenoyl)-sphing-4-enine-1-phosphocholine (NSM); 1-palmitoyl-2-linoleoylphosphatidylcholine (PLPC); 3-palmitoyl-2-oleoyl-d-glycero-1-phosphatidylethanolamine (POPE); 1-palmitoyl-2-linoleoyl-phosphatidylethanolamine (PLPE); 1-palmitoyl-2-linoleoyl-phosphatidylserine (PLPS).

Upper Leaflet		Lower Leaflet	
Lipid	Relative Level	Lipid	Relative Level
CHOL	11	CHOL	11
SOPC	6	SOPC	3
POPC	4	POPE	2
SSM	2	POPC	2
NSM	1	PLPE	2
PLPC	1	PLPS	2

A common pose was apparent for all three ligands, with the conserved oxindole moiety wedging between γ8 M3_T_ and M4_T_ at the level of Val-176 and Gly-209. In this binding mode, an amine of the isostere acts as a proton donor (Fig. 2*A*), H-bonding with the γ8 Asn-172 side-chain oxygen (O_δ1_) (Fig. 3*A*). These two features are consistent with the published structure activity relationship (SAR) and with mutagenesis studies where potency dropped dramatically in the γ8 N172A mutant (ΔpCI_50_ > 2; *i.e.* two orders of magnitude (10)). We noted two deviations from this predominant arrangement. For JNJ-059, stacking interactions between the variable region of the compound and γ8 Phe-212 resulted in a tilt of the oxindole in the binding pocket and a loss of H-bonding with Asn-172. In a separate docking pose, for LY-481 we also observed engagement of the variable region's hydroxyl group with the pocket, which appears unlikely when considering the published SAR data, highlighting the importance of the oxindole moiety for function (Fig. S1, *B* and *C*).

### Analyzing ligand binding by induced-fit docking

To extend these observations we next performed induced-fit dockings using Glide (Schrödinger, LLC, 2019 (42)) and modeled surrounding residues with PRIME (43). In this configuration, side chains of the receptor can dynamically adjust around the ligand during binding (43). Mirroring the results obtained with rigid docking, for all three modulators we consistently observed engagement of the Val-176–Gly-209 pocket by the oxindole moiety and H-bonding between the ligands and the Asn-172 side chain (Fig. 3*B*).

Induced-fit docking revealed another orientation of the LY-481 variable region, which projected toward the outer leaflet surface (Fig. 3*B1*), rather than into the lipid bilayer (Fig. 3*A1*). This alternative conformation is facilitated by the flexible linker of the modulator (Fig. 2*A1*, *green*) and could be energetically more favorable because the ligand is able to engage the upper part of the γ8 M3_T_ and M4_T_ helices, the AMPAR M1 helix, and water molecules at the membrane/solvent boundary. Interestingly, this pose could explain the different activity profiles of the (R)- and (S)-enantiomers of LY-481 (12): the methyl group of the (R)-enantiomer would clash with the Cα of Gly-209 and thus be less favorable than the (S)-isomer (Fig. S3, *A* and *B*). Our docking studies provide a first view of how the three modulators are likely to dock to the γ8 specific Val-176/Gly-209 pocket through their oxindole isostere.

### Monitoring modulator engagement of TARP γ8 in MD simulations

**Figure 4.**
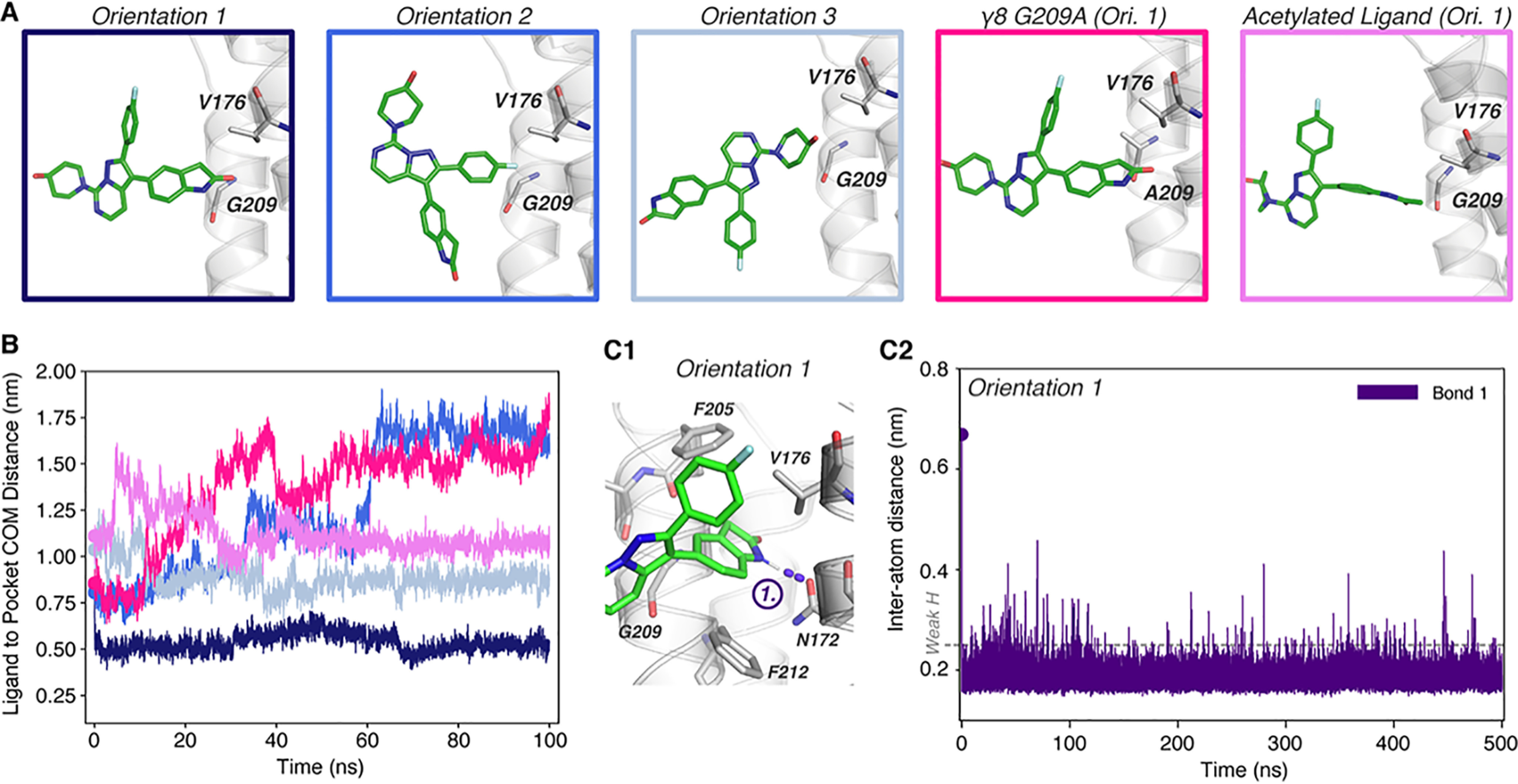
**Molecular dynamics simulation of JNJ-61432059 binding to TARP γ8**. *A*, initial orientations and positions of JNJ-059 at simulation initiation, near the binding pocket between γ8 Val-176 and Gly-209 (*sticks*). Orientation 1 was simulated with the TARP G209A mutation (mimicking other type I TARPs), or the inactive JNJ-059 precursor, which contains an acetylated aniline instead of its oxindole group (acetylated ligand). *B*, measurement of the distance between the COM of the ligand and the binding site (Cα-atoms of Gly-209 and Val-176) demonstrates the binding behavior of JNJ-059 from simulations, indicating successful binding only in orientation 1. *C1*, pose from simulation of orientation 1 at 200 ns, depicting formation of a hydrogen bond (*purple*) between the oxindole group of JNJ-059 and Asn-172 of γ8. *C2*, measurement of the distance between the bonding hydrogen of JNJ-059 oxindole and the residual oxygen of Asn-172 shows rapid and stable formation of a hydrogen bond (<0.25 nm required for weak H-bond, *dotted line* (68)) throughout the simulation.

To gain insight into the stability of a given binding pose over time, we next conducted MD simulations to explore if spontaneous binding is observed when ligands are placed close to their binding site. In contrast to the docking algorithms, MD simulations explicitly consider the presence of solvent and lipids (Table 2), which provides a realistic environment for a transmembrane binding site. To reduce bias, we placed the ligands in various orientations in front of the γ8 pocket (Fig. 4*A* for JNJ-059), avoiding any steric clashes or pre-docking in the pocket. Ligand engagement was monitored by computing the distance between the center of mass (COM) of a given ligand with the center of the Val-176/Gly-209 Cα atom pair.

### JNJ-059

This analysis is first shown for JNJ-059 (Fig. 4). Because JNJ-059 has three distinct groups extending from a “central point” (Fig. 2*A3*), three orientations of the keto tautomer were considered (Fig. 4*A*). Only orientation 1, where the oxindole isostere group is closest to the binding site, resulted in stable binding (Fig. 4*B*), and an H-bond between the oxindole nitrogen and the Asn-172 side chain (*bond 1*; Fig. 4*C1*) was established in the first 400 ps and then maintained throughout the 500-ns simulation (Fig. S4*A* and Fig. 4*C2*). The pose of the ligand (Fig. 4*C1*) closely mirrors the binding mode from our docking results. The characteristic tilt of the oxindole group in the binding site was replicated and accounts for the distance drop during the simulation at ∼70 ns (Fig. 4*B*, *orientation 1*). A 200-ns repeat run reproduced this behavior (Fig. S4*B*). We also tested binding to the G209A γ8 mutant in the same orientation, where the H-bond to Asn-172 formed in the first 3 ns but the ligand was unable to penetrate into the pocket and dissociated (Fig. 4, *A* and *B*). The validity of our predicted docking mode was further tested by simulating the inactive acetylated aniline derivative of JNJ-059 (Fig. S1*J*) (13), which was also unable to stably engage the binding site (Fig. 4, *A* and *B*).

### JNJ-118

**Figure 5.**
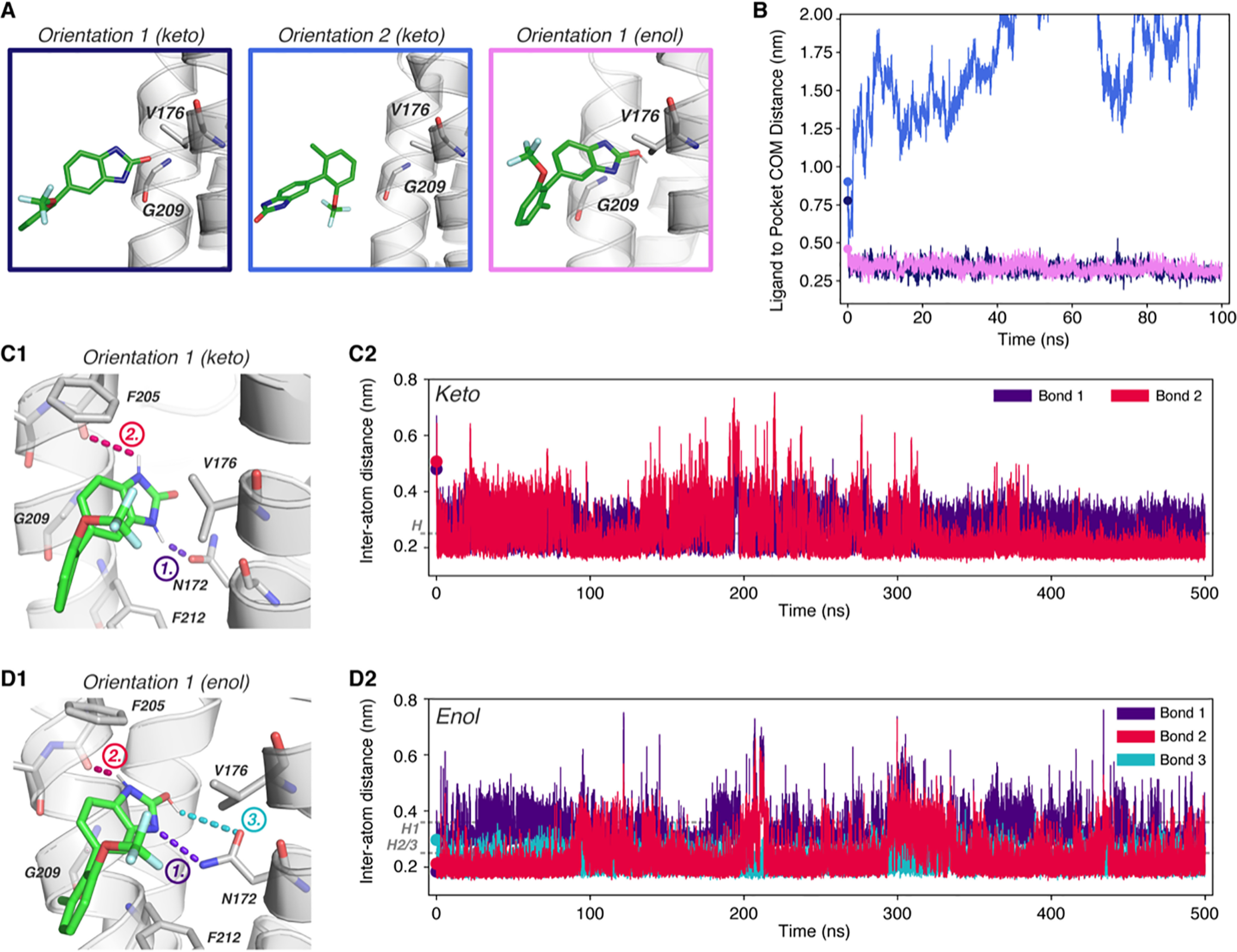
**Molecular dynamics simulation of JNJ-55511118 binding to TARP γ8**. *A*, JNJ-118 initial orientations outside the γ8 binding pocket between Val-176 and Gly-209 (*sticks*). Orientation 1 was simulated with the enol form of JNJ-118. *B*, measurement of the ligand and binding site (Cα-atoms of Gly-209 and Val-176) COMs demonstrating specific binding of JNJ-118 in orientation 1 and successful binding of both JNJ-118 tautomers. *C1*, pose at 200 ns from simulation of orientation 1 (keto form JNJ-118) depicting formation of hydrogen bonds with γ8 through the backbone of Phe-205 (*red*) and side chain of Asn-172 (*purple*). *C2*, measurement of the distance between bonding atoms of the two hydrogen bonds throughout the simulation, demonstrating stable bond formation (colors as per *C1*). *D1*, binding pose at 200 ns of the JNJ-118 enol tautomer, which forms three hydrogen bonds with residue Phe-205 (backbone, *red*) and two with Asn-172 (*purple* and *blue*). *D2*, bonding analysis by distance measurement between bonding atoms from *D1* shows stable bond formation. Note, JNJ-118 oxindole nitrogen to Asn-172 bond measurement (*purple*) is from N to N atom, rather than N to H, meaning successful bond formation requires a distance of <0.36 nm in this instance (68).

For JNJ-118, the chlorine atom in the variable region of the ligand (Fig. 2*A2*) prevented parametrization by the CHARMM General Force Field (44) and was therefore replaced by a methyl group; although this variety has not been tested experimentally, a methyl substitution (Fig. S1*F* versus Fig. S1*G*) still functioned as an effective γ8 modulator (35). Of two orientations tested, only orientation 1 resulted in stable binding that was maintained throughout a 500-ns simulation (Fig. 5, *A* and *B*, and Fig. S4*C*). The two nitrogen atoms of the benzimidazolone moiety engaged γ8 through two H-bonds, one with the Asn-172 side chain (O_δ1_; bond 1) and the other with the Phe-205 main chain carbonyl (bond 2) (Fig. 5*C1*). Whereas bond 2 was more stable in one simulation (Fig. 5*C2*), bond 1 was more persistent in a second run (Fig. S4*D1*), and no ligand binding was seen in run 3 (not shown), implying greater flexibility of this ligand. We also investigated the enol tautomer (Fig. 5, *D1* and *D2*), which adopted a comparable pose but formed a third H-bond between its hydroxyl group and the oxygen of the Asn-172 side chain (O_δ1_; bond 3), whereas the deprotonated nitrogen atom changed its hydrogen bond partner to the nitrogen of the Asn-172 side chain (N_δ2_). All these three bonds persist through a 500-ns simulation and in repeat runs, although all of them exhibit some distance fluctuations (Fig. 5*D2* and Fig. S4*D2*). The binding poses for both JNJ-118 isomers are shown in Fig. 5, *C1* and *D1*.

### LY-481

**Figure 6.**
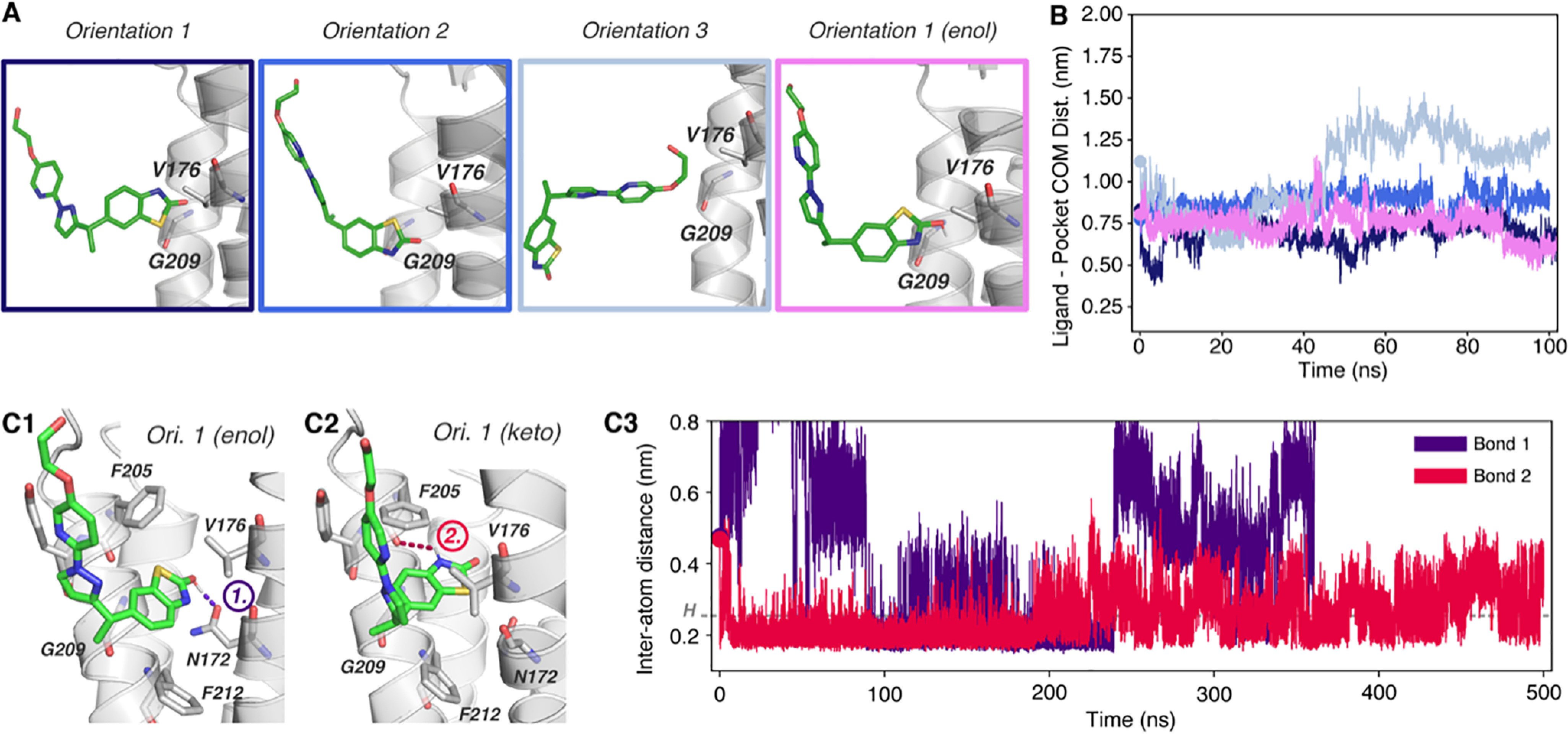
**Molecular dynamics simulation of LY-3130481 binding to TARP γ8**. *A*, LY-481 initial orientations outside the γ8 binding pocket between Val-176 and Gly-209 (*sticks*). Orientation 1 was also simulated with the LY-481 enol form. *B*, measurement of the ligand and binding site (Cα-atoms of Gly-209 and Val-176) COMs demonstrating binding of LY-481 in orientation 1, but not orientations 2 and 3. The enol form also binds late in the simulation (after 80 ns). *C1–C2*, binding poses at 200 ns from simulation of keto (*C1*) and enol (*C2*) tautomers of LY-481 in orientation 1 depicting differential hydrogen bond formation. The keto form hydrogen bonds with the Phe-205 backbone (*red*), whereas the enol form bonds with the Asn-172 side chain (*purple*). *C3*, measurement of the distance between bonding atoms in final poses demonstrates the duration of hydrogen bond formation during the simulation. Note that the distance of bond 1 is larger than 0.8 nm after 350 ns.

For LY-481, in addition to the three orientations used for the other compounds, we also considered a rotation of the benzothiazolone moiety because the sulfur atom in this group could engage the Asn-172 side chain (orientations 1 *versus* 2) (Fig. 6*A*). Contrasting with the two JNJ ligands, LY-481 exhibited a more heterogeneous binding behavior, possibly reflecting its reduced pIC_50_ value (Fig. S1). Engagement of the keto form with the pocket was mainly observed when the ligand's sulfur atom pointed toward Asn-172 (orientation 1) and not when facing Phe-205 (orientation 2). In orientation 2, the modulator approaches Gly-209 but does not proceed fully into its binding site (Fig. 6, *A* and *B*, and S4*E*). The keto isomer resided in the pocket nearly throughout the 500-ns simulation, although the H-bond to Phe-205 is less stable in the second half of the simulation (*bond 2*, Fig. 6*C3***)**. Interestingly, in one of the repeat runs, we also observed flipping of the benzothiazolone group within the binding site, where the bond switches from Phe-205 to H-bond with Asn-172 (Fig. S4*F1*).

Finally, we investigated the enol tautomer, which we hypothesize to be the major isoform of LY-481 (Fig. 2*B1*). To mimic poses observed with other ligands more closely, we orientated the sulfur of the benzothiazolone toward Phe-205, resulting in apposition of the ligand's amine with Asn-172 (Fig. 6, *A* and *C1*). Binding of the enol was also heterogenous and we observed three different behaviors: a binding event at ∼90 ns and ligand disengagement at ∼230 ns (*bond 1*; Fig. 6*C3*); indirect binding to Asn-172 via a water molecule, thus increasing the inter-atom distance (*replica 2*; Fig. S4*F2*); and the ligand does not engage the pocket (*replica 3*; Fig. S4*F2*). As observed with induced-fit docking (Fig. 3*B1*), the variable region tail of the molecule projects toward the outer leaflet where it could interact with either M3_T_ or M4_T_ (Fig. S3*C*). As noted above, this is the only pose accounting for the high potency of the (S)- *versus* the (R)-enantiomer (Fig. S3, *A* and *B*).

In orientation 4, we replicated a binding mode described for LY-481 based on a claudin-19 homology model (36), which we could not reproduce (see supplementary discussion) (Fig. S5).

### Functional comparison of LY-481 and JNJ-118

**Figure 7.**
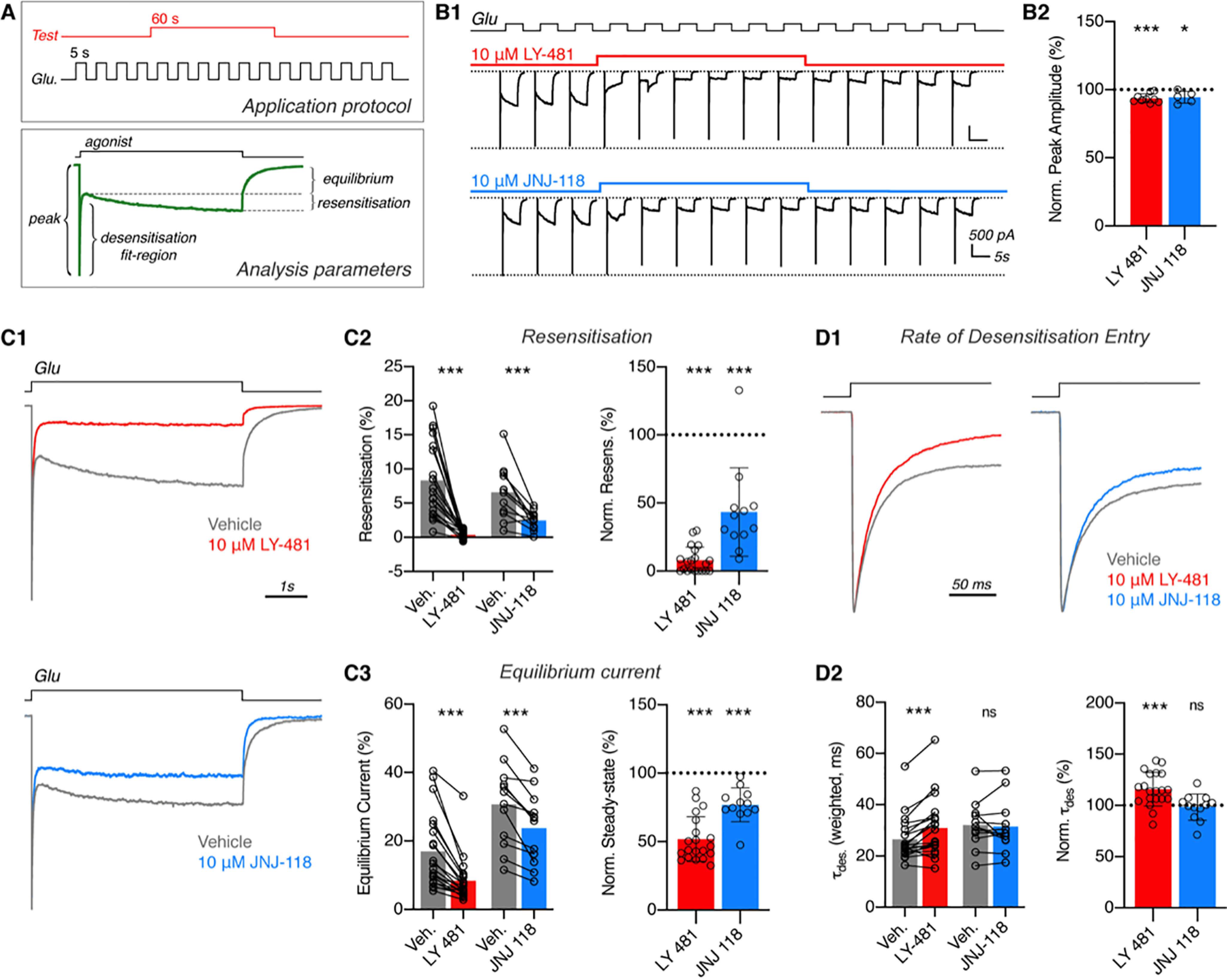
**Characterization of TARP γ8 modulatory compounds on recombinant AMPAR responses.** *A*, drug application protocol schematic (*top*). Test compounds (*red*) were continuously co-applied with 5-s applications of 10 mm glutamate (AMPAR agonist). Multiple components of the resulting current responses (*green example trace*, *bottom*) were compared on drug application, as depicted. *B1*, example traces of glutamate responses from GluA2_γ8 expressing lifted whole cells with a 10 µm application of modulatory compounds as indicated. Some washout of response antagonism can be seen. *B2*, peak response amplitudes were marginally reduced by compound application (normalized to pre-application responses) (LY-481: 93.6 ± 3.3%, *n* = 9 cells, *p* = 0.0004; JNJ-118: 94.5 ± 4.3, *n* = 5 cells, *p* = 0.046; one sample *t* test *versus* 100%). TARP γ8 modulatory compounds both attenuate resensitization (*C2*; *LY*, *vehicle*: 8.31 ± 5.40%, LY-481: 0.38 ± 0.59%, *n* = 20 cells, *p* < 0.0001; *JNJ*, *vehicle*: 6.60 ± 3.91%, JNJ-118: 2.48 ± 1.31%, *n* = 12 cells, *p*= 0.001; Wilcoxon tests) and steady-state amplitude (*C3*; *LY*, *vehicle*: 16.96 ± 11.02%, LY-481: 8.35 ± 6.65%, *n* = 20 cells, *p* < 0.0001; *JNJ*, *vehicle*: 30.72 ± 12.18%, JNJ-118: 23.77 ± 10.47, *n* = 12 cells, *p* = 0.0005; Wilcoxon tests) of AMPAR responses, as seen from example traces with normalized peak amplitudes (*C1*). *C2* and *C3*, paired data depict actual values for individual cells pre- and post-modulatory compound application, and data normalized to pre-application responses is presented *right* (*Normalized Resensitization*, LY-481: 8.0 ± 9.6%, *p* < 0.0001; JNJ-181: 43.3 ± 32.5%, *p* < 0.0001, One sample *t* test *versus* 100%; *Normalized Equilibrium*, LY-481: 51.7 ± 16.3%, p < 0.0001; JNJ-118: 76.9 ± 12.4%, *p* < 0.0001; One sample *t* test *versus* 100%). *D*, rate of desensitization entry does not show loss of TARP γ8 modulation by either compound (peak normalized examples traces, *D1*). Rate of desensitization entry is slowed by LY-481 (Weighted tau; *vehicle*: 26.50 ± 8.64 ms, LY-481: 30.88 ± 11.54 ms, *n* = 20 cells, *p* = 0.0002, Wilcoxon test), but not by JNJ-118 (*vehicle*: 32.06 ± 9.27 ms, JNJ-118: 31.48 ± 10.42 ms, *n* = 12 cells, *p* = 0.91, Wilcoxon test) (*D2*). Data normalized to pre-application kinetics are depicted (*right*) (LY-481: 115.6 ± 16.3%, *p* = 0.0004; JNJ-118: 98.3 ± 12.8%, *p* = 0.66; one-sample *t* tests *versus* 100%). All data are reported as mean ± S.D.

Our docking data point to a common mode of TARP γ8 engagement by three structurally distinct NAMs. Given this similarity, we wanted to directly compare the functional profile of the modulators, which could have similar effects. Hence, we compared the two originally reported ligands, LY-481 and JNJ-118, side by side on recombinantly expressed AMPARs by patch-clamp recordings (Fig. 7). The GluA2 subunit (**Q**/R, **R**/G, **flip**/flop), fused at its C terminus to γ8, in the tandem configuration (45) to ensure full receptor occupancy with a TARP (termed GluA2_γ8, see “Experimental procedures”), was expressed in HEK293T cells for fast-agonist application whole-cell recordings. TARP γ8 has multiple effects on the kinetics of the AMPAR, predominantly slowing the rate of desensitization and consequently increasing the magnitude of equilibrium currents (46, 47).

We used a 5-s agonist application protocol (Fig. 7*A*), which permits analysis of multiple kinetically distinct components of the AMPAR response, including the rate of entry into desensitization, an agonist-bound nonconducting state, and allows sufficient time to observe resensitization, a TARP-induced state that is characterized by a gradual increase in current amplitude after initial desensitization (25, 48). Resensitization is unique to AMPARs associated with TARPs γ4, γ7, and γ8 and requires full TARP occupancy (*i.e.* four TARP molecules per AMPAR tetramer) (25, 49). Although this component has previously been reported to be blocked by LY-481 (11, 36), the effect of JNJ-118 on resensitization is currently unknown.

**Figure 8.**
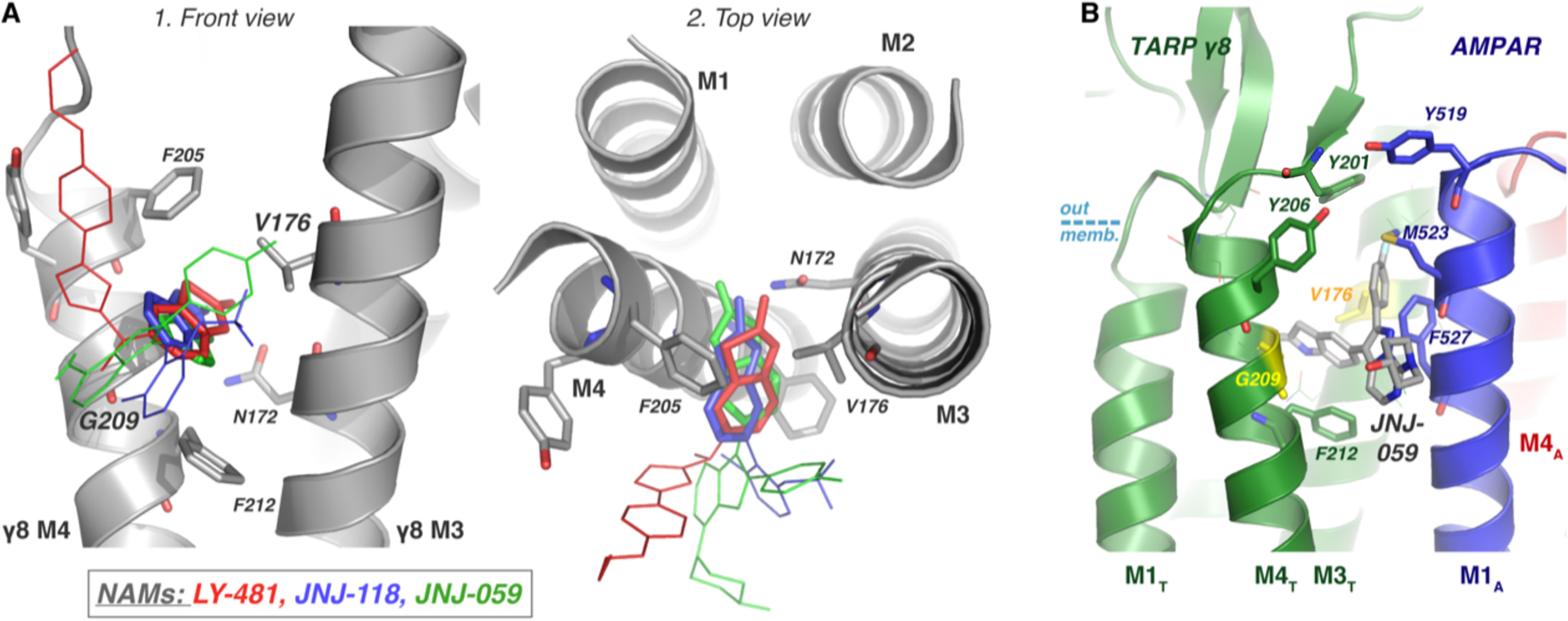
**Summary of predicted binding modes.** *A*, the three AMPAR-TARP NAMs (LY-481, *red*; JNJ-118, *blue*; JNJ-059, *green*) all have a similar binding mode in TARP γ8, as depicted on the modeled protein structure, facilitated by γ8 specificity residues Gly-209 and Val-176, and stabilized by Asn-172. The oxindole isostere moiety, conserved between the three ligands, are shown in *bold*, and the variable regions are depicted as *thin lines*. *B*, the pose shown in Fig. 4*C1* was placed in the context of the TARP/AMPAR complex (PDB 6QKC) with minor changes. As shown, the ligand predominantly interferes with interactions between M4_T_ and M1_A_. AMPAR residues Phe-527 and Met-523, which contact TARP in the cryo-EM structure, and TARP tyrosines capping the pocket (Tyr-201 and Tyr-206) may be primary contact points. We note that the exact pose of the variable position is currently unclear.

After obtaining stable glutamate-gated AMPAR responses, TARP modulators were applied for at least 60 s to achieve complete modulation (Fig. 7, *A* and *B*). Neither compound had a major effect on the magnitude of peak currents as had previously been suggested (10), both subtly reducing their amplitude by less than 10% (Fig. 7*B*), with no effect on their rise time (Fig. S7). Modulator application rapidly suppressed both resensitization (Fig. 7, *B1*, *C1*, and *C2*) and the magnitude of the equilibrium current (Fig. 7, *B1*, *C1*, and *C3*), with LY-481 showing greater NAM efficacy on both parameters. To more directly compare between ligands, currents were normalized to pre-application conditions within each recording, demonstrating that LY-481 reduced resensitization to 8.2 ± 2.1% *versus* 43.3 ± 9.4% for JNJ-118, whereas steady-state currents were reduced to 51.7 ± 2.6% for LY-481 and to 76.9 ± 3.6% for JNJ-118 (Fig. 7, *C2* and *C3*, *right panels*). A hallmark of TARPs (including γ8) is a slowing of the entry rate into desensitization (17, 18). We did not observe negative modulation of this component as could be expected by a block of TARP modulation (*i.e.* ligand-induced speeding of desensitization), and conversely observed subtly decelerated desensitization kinetics, specifically for LY-481. It is possible that this effect manifests in the ensemble current as a result of resensitization inhibition. Taken together, our comparative analysis demonstrates analogous functional effects of the modulators, which may result from their comparable binding mode (Fig. 8), but also reveals some differences in their functional influence, likely deriving from the structural variations between the modulators. Based on these data, the primary effect of these NAMs can be attributed to their blunting of the resensitization and equilibrium current components.

## Discussion

This work sheds light onto the workings of structurally diverse TARP γ8 modulators, which have great potential to be clinically important AMPAR therapeutics. A combination of *in silico* approaches point to a main binding pose involving an oxindole isostere substructure that is common to all three compounds, despite their identification from independent screening projects (10, 12, 13). This moiety of the ligand wedges between the AMPAR-interacting M3_T_ and M4_T_ helices of γ8 between Val-176 (in M3_T_) and Gly-209 (in M4_T_), the residues that confer selectivity over other TARPs (Fig. 8*A*). A crucial role for this moiety has been confirmed by extensive SAR data, supporting its importance for binding (12, 13, 35). Within the pocket, the planar oxindole isostere of the three ligands locates mostly with a comparable tilt angle and can form a hydrogen bond with the γ8 Asn-172 side chain, stabilizing its binding; mutation of this residue has previously demonstrated its importance for modulator action (10, 11). The angle of the oxindole ring system is determined by the width of the binding pocket, *i.e.* the level of separation between M3_T_ and M4_T_, which our MD simulations suggest can dilate (Fig. S2). The AMPAR-γ8 cryo-EM complex had insufficient separation of these helices for any of the ligands to bind (PDB 6QKC), with a Cα distance between Val-176 and Gly-209 of 8.5 Å (34). The MD simulations showed expansion to 9.4 Å (AMPA/γ8 complex) or 11.7 Å (γ8-only model), which is partly due to a penetration of water molecules toward the pocket (Fig. S2). Docking poses from the AMPAR/γ8 complex were more consistent between the three ligands compared with the γ8-only simulations, suggesting that even a subtle dilation of the γ8 M3_T_ and M4_T_ helices suffices for ligand binding. As γ8 itself can bind to a radiolabeled LY-481 derivative in HEK293 cells (36), free γ8 was an appropriate subject for our studies.

The orientation of the structurally diverse variable region and its interactions with the receptor are currently unclear for both JNJ compounds because multiple conformations were observed and will need to be resolved through structural studies. Given the conserved binding mode of the oxindole motif, the interactions of the variable region likely contribute to the functional differences described here. A possible binding mode for JNJ-059 is presented in Fig. 8*B*, which highlights residues on the AMPAR (M1_A_) that are likely contacted by the modulator. These residues are Tyr-519, Met-523, and Phe-527 (in GluA1), in a region adjacent to the M3 channel gate and proximal to residues that have been implicated in gating modulation (34, 50). All proposed poses could also decrease packing between the receptor and γ8, specifically between M4_T_ and M1_A_. Met-523 (GluA1) is in close contact with γ8 in PDB 6QKC, and any modulator likely clashes with this residue in the common binding pose. Of note, we also used two approaches to analyze ligand interactions with the AMPAR-TARP complex but with inconclusive results. First, induced-fit docking, although it restrained the ligand by an H-bond to γ8 Asn-172, yielded no reasonable structure. We hypothesize that the induced-fit algorithm could not deal with the hydrophobic environment outside the pocket accurately and hence forced receptor side chains into unrealistic positions. Secondly, the ligand was docked into TARP γ8 in a 200-ns MD run, which was then placed opposite the AMPAR (while avoiding clashes). However, when pulling the proteins together by force-probe MD, we did not obtain a reasonable structure.

For LY-481, an orientation directed toward the outer membrane surface was apparent in induced-fit docking and in MD simulations. This pose permits favorable interaction of the variable ligand region with M3_T_ and M4_T_ specifically for the (S)-enantiomer, which could explain greater potency of this isomer (12). Considering the ligand environment of this pose might facilitate structure-based development of more potent LY-481 derivatives. Interactions with either Asn-183 or Tyr-201 of γ8, which are located at the boundary between the membrane and the exterior milieu, may point to an entry route for LY-481 to the binding site at the AMPAR-TARP interface (Fig. S3*C*).

Based on NMR data, the oxindole isosteres of JNJ-118 (a benzimidazolone) and LY-481 (a benzothiazolone) also exist as enol tautomers, which feature a similar binding mode overall but exhibited different H-bond stabilities (68), especially to Asn-172. The rate of H-bond formation and breakage imply a geometric component altered by the enol tautomer because it places its hydrogen donor deeper in the pocket. However, calculating H-bond energies from simulations requires further validation of ligand parametrizations. For JNJ-118, the enol can form an additional H-bond with Asn-172-O_δ1_ through its terminal hydroxyl, producing in total three H-bonds compared with two for the keto isomer, which could increase the affinity of the enol tautomer (Fig. 5, *C1* and *D1*). Because the equilibrium of these two isomers could not be differentiated from NMR spectra, co-existence with the less potent keto tautomer could potentially compromise the affinity of JNJ-118. The lower potency of JNJ-118, relative to JNJ-059, might also be caused by the deeper penetration of JNJ-059 into the binding pocket and is evident from its heterogeneous behavior in MD simulations. This dichotomy between tautomer H-bonding patterns and penetration into the pocket is also apparent for LY-481. The overall heterogeneous behavior of this ligand may reflect its relatively lower affinity; therefore, based on these data it is difficult to determine which isomer binds more favorably (Fig. S1). Although the enol penetrated more deeply into its binding site (Fig. S6 at ∼200 ns), H-bonding and residence in the pocket were less stable compared with the keto tautomer. It is also worth noting that, unlike the keto isomer, the enol form approaches the pocket from a distance and “finds” its pocket at ∼90 ns, indicative of a realistic binding event (Fig. 6*C3*).

Functional comparison of JNJ-118 and LY-481 on GluA2_γ8 provide additional insight because these ligands have yet to be tested comparatively. Overall, both NAMs have very specific effects on the AMPAR current response and do not prevent all components of TARP γ8 modulation, such as desensitization kinetics. The fact that certain facets of TARP modulation remain intact in the presence of the NAMs is a further indication that these ligands do not physically displace γ8 from the AMPAR, in line with earlier studies (10, 11, 36). The main NAM effects observed were a block of resensitization and, to a lesser extent, a reduction in equilibrium current magnitudes. Whereas the complete inhibition of resensitization may contribute to the effect on steady-state, other kinetic parameters such as the rate of recovery from desensitization, which we have not assessed, may also contribute to this effect. LY-481 modulated both parameters more effectively, despite forming only one H-bond (with Asn-172-O_δ1_ in the predominant enol form). This may indicate that the variable region of the ligand, which is more substantial in LY-481 and engages γ8 M3_T_ and M4_T_ (Fig. 3*B1*), has a major influence on ligand potency. Given that the mechanism by which TARPs act to produce resensitization is not yet understood (25, 48, 51), we are unable to interpret how the binding of modulators at this site can prevent the action of TARPs. Resensitization, similarly to NAM binding, is specific to TARP γ8 over other TARP family members, and whether these observations are related would require further study. Future structure-function relationship studies will undoubtedly clarify how these promising modulators exhibit their effects on γ8-containing AMPARs.

## Experimental procedures

### Structural modeling with MODELLER

An all-atom model of the AMPAR (GluA1/GluA2 heteromer) in complex with TARP γ8 was created based on the published cryo-EM structure PDB 6QKC, which has a resolution of 4.4 Å (34). Not all residues are resolved in this structure; therefore, the corresponding *Rattus norvegicus* TARP γ8 sequence was obtained from UniProt (Q8VHW5) for model completion. Only five residues differ between *Homo sapiens* and *Rattus norvegicus* TARP γ8 protein sequences (PSI-BLAST (52)) in the range modeled (Met-1–Leu-241), and with no differences in the AMPAR-interacting helices, no difference in ligand binding is expected between rat and human TARP γ8. As the TARP γ8 C terminus is apparently disordered and likely not to influence ligand binding, residues after Leu-241 were not modeled. For modeling of missing atoms, MODELLER version 9.22 was used (38, 53, 54). To increase accuracy of the *ab initio* modeling of the disordered extracellular TARP loops, the MD refinement was set to “slow” and the optimization protocol was repeated 10 times. 10 models were output with MODELLER's DOPE score describing the energy of system, where lower energy reflects a higher quality model (39). The RMSD of atom positions between the models and the cryo-EM structure 6QKC was calculated as another measure of reliability using PyMOL (Schrödinger, LLC, 2015). Model 8 was selected as the most reasonable, having the lowest energy based on the DOPE score and the smallest RMSD from the template structure (see Table 1).

### Molecular dynamics simulation

Simulation setup was performed with CHARMM-GUI v1.7 (55, 56), using the MODELLER-generated model 8 as input. Lipids were chosen based on previous simulations of GluA2 in a heterogeneous lipid bilayer (57), from which the six most abundant lipids in the lower and upper leaflets were chosen. Lipid stereochemistry was further simplified by rounding the fractions reported previously (57). This enabled a small simulation box. If the lipids reported were not found in CHARMM-GUI (56), they were changed, while maintaining the head group and number of unsaturated bonds. The resulting composition of the lipid bilayer is reported below in Table 2. If ligands were used in the simulation, they were built and minimized in the Schrödinger Suite Maestro (Schrödinger, LLC, 2019) to ensure a proper geometry. Afterward, the ligands were parametrized by the CHARMM General Force Field program version 2.2.0 (44, 58). Simulations were performed in 150 mm sodium chloride placed by the Monte-Carlo method of CHARMM-GUI, in addition to the ions for neutralizing the system. The protonation state of all amino acids corresponded to pH 7. The water model used was transferable intermolecular potential with three points (59), and the simulation temperature was 310.15 K. The pressure was maintained at 1 bar. To maintain the temperature, a Nosé–Hoover temperature coupling method (60) with a tau-t of 1 ps was used, and for pressure coupling, a semi-isotropic Parrinello–Rahman method (61) with a tau-p of 5 ps and a compressibility of 4.5 × 10^−5^ bar^−1^ was used. The equilibration protocol was performed according to the standards of CHARMM-GUI (55). The CHARMM36m force field was used (62). The simulation was computed using GROMACS 2019.3 (63, 64).

### Docking

The Schrödinger LigPrep (65) utility was run using default parameters at pH 7.0 to prepare ligands for docking. Rigid docking was performed with AutoDock Vina version 1.1.2 (40). The docking was performed using the aforementioned model in a 60 × 60 × 20 Å box with center of mass between TARP residues Gly-209 and Val-176. The exhaustiveness was set to 100 and 20 poses were produced. In this algorithm, the protein is rigid, but the ligand remains flexible. The induced-fit docking protocol of Schrödinger was used to model protein adaption upon ligand binding (43), using the same center of mass to define the docking box. Extended sampling parameters were used such that after initial docking with Glide (42), the residues within 5 Å of the ligand were modeled with full flexibility using Prime (66), generating up to 80 complexes per ligand.

### cDNA constructs

GluA2 (rat cDNA sequence, flip isoform, R/G edited, Q-pore) was expressed in a tandem configuration (denoted GluA2_γ8) with TARP γ8 (rat cDNA sequence) by cloning the TARP γ8 coding sequence (Glu-2–Val-423) at the extreme C terminus of the GluA2 coding sequence, in the pRK5 vector, separated by a Gly-Ser-Gly-Ser-Gly linker sequence, using *in vivo* assembly cloning (67). pN1-EGFP (Clontech) was used for visualization of transfected cells.

### Electrophysiology

HEK293T cells (ATCC cat no. CRL-11268, RRID: CVCL_1926, lot 58483269; identity authenticated by short tandem repeat analysis, mycoplasma negative), cultured at 37 °C and 5% CO_2_ in DMEM (Gibco; high glucose, GlutaMAX, pyruvate, cat no. 10569010) supplemented with 10% fetal bovine serum (Gibco) and penicillin/streptomycin, were transfected using Effectene (Qiagen) according to manufacturer protocol. GluA2_γ8 and EGFP plasmids were transfected at a 9:1 stoichiometry to aid identification of AMPAR-containing cells. 36 h after transfection, cells were split using a brief EDTA wash and plated on poly-l-lysine–coated glass coverslips on the morning of recording. 30 µm 2,3-dioxo-6-nitro-1,2,3,4-tetrahydrobenzo[f]quinoxaline-7-sulfonamide (Tocris) was added to media post-transfection to avoid AMPAR-mediated toxicity.

Lifted whole cells were held in the whole-cell patch-clamp configuration, voltage clamped at −60 mV, and subjected to fast application of 10 mm l-glutamate using a two-barrel theta glass tube controlled by a piezoelectric translator (Physik Instrumente), allowing solution exchange in around 200 μs. Signals were acquired using the MultiClamp 700B amplifier (Axon Instruments), digitized using a Digidata 1440A interface, and recorded with pClamp10 (Molecular Devices). Extracellular solution contained (in mm) NaCl (145), KCl (3), CaCl_2_ (2), MgCl_2_ (1), glucose (10), and HEPES (10), adjusted to pH 7.4 using NaOH. Borosilicate glass electrodes (1.5 mm outer diameter, 0.86 mm inner diameter, Science Products GmbH), pulled with a PC-10 vertical puller (Narishige) with tip resistance of 2–5 MΩ, were filled with internal solution containing (in mm) CsF (120), CsCl (10), EGTA (10), ATP-sodium salt (2), HEPES (10), and spermine (0.1), adjusted to pH 7.3 with CsOH. Correction was not made for the liquid junction potential.

During application, a ligand was constantly present both during and between glutamate pulses. Modulatory compounds were made up to 50 mm stock solutions in DMSO and used at a final concentration of 10 µm throughout. On the developer's instructions, JNJ-55511118 (Tocris) was brought into recording solution by addition of 1:1 JNJ-55511118 stock solution with 10% Pluronic F-127 (Thermo Fisher Scientific) before gradual addition of extracellular recording solution while vortexing. Vehicle solutions were made up in an equivalent manner, using DMSO and 10% Pluronic F-127. LY-3130481 (custom synthesis, according to the published procedure (12)) was solubilized in recording solution either in the same manner as above, or by addition of DMSO stock to final volume of extracellular recording solution (without Pluronic F-127). No difference in solubilization or drug efficacy was exhibited between solubilization methods, and therefore results were combined.

Agonist was applied to lifted whole cells in 5-s pulses every 10 s. Test compounds were applied for at least 60 s using the two-barrel theta glass applicator. Coverslips were exchanged after every successful recording to prevent pre-exposure of cells to modulatory compounds prior to recording. Desensitization entry was determined from the first 200 ms after the peak response, which was fitted with a two-exponential function to obtain the (weighted) time constant. Steady-state responses were denoted as the percentage of peak current remaining after 200 ms. Resensitization is determined as the percentage of peak current that recovers between 200 ms and 5 s of glutamate application:

$$Steady-state\;current=\left(I_{200\;ms}\right)/\left(I_{peak}\right)\;\times \;100\%$$

Resensitization=(I5 s− I200 ms)/(Ipeak) × 100%

Changes in peak current were only quantified from cells where the peak current was stable for at least three consecutive sweeps, to avoid rundown-induced misinterpretation.

## Data availability

The data generated and analyzed in this study are included in this article and in the supporting information, or can be obtained from the authors upon reasonable request.

## Supplementary Material

Supporting Information

## References

[1] Traynelis S. F., Wollmuth L. P., McBain C. J., Menniti F. S., Vance K. M., Ogden K. K., Hansen K. B., Yuan H., Myers S. J., and Dingledine R. (2010) Glutamate receptor ion channels: structure, regulation, and function. Pharmacol. Rev. 62, 405–496 10.1124/pr.109.002451 20716669 PMC2964903

[2] Kessels H. W., and Malinow R. (2009) Synaptic AMPA receptor plasticity and behavior. Neuron 61, 340–350 10.1016/j.neuron.2009.01.015 19217372 PMC3917551

[3] Bowie D. (2008) Ionotropic glutamate receptors & CNS disorders. CNS Neurol. Disord. Drug Targets. 7, 129–143 10.2174/187152708784083821 18537642 PMC2662616

[4] Lynch G. (2004) AMPA receptor modulators as cognitive enhancers. Curr. Opin. Pharmacol. 4, 4–11 10.1016/j.coph.2003.09.009 15018832

[5] Partin K. M. (2015) AMPA receptor potentiators: from drug design to cognitive enhancement. Curr. Opin. Pharmacol. 20, 46–53 10.1016/j.coph.2014.11.002 25462292 PMC4318786

[6] Ishii T., Stolz J. R., and Swanson G. T. (2020) Auxiliary proteins are the predominant determinants of differential efficacy of clinical candidates acting as AMPA receptor positive allosteric modulators. Mol. Pharmacol. 97, 336–350 10.1124/mol.119.118554 32111699

[7] Rogawski M. A. (2013) AMPA receptors as a molecular target in epilepsy therapy. Acta Neurol. Scand. 127, 9–18 10.1111/ane.12099 PMC4506648 23480151

[8] Balannik V., Menniti F. S., Paternain A. V., Lerma J., and Stern-Bach Y. (2005) Molecular mechanism of AMPA receptor noncompetitive antagonism. Neuron 48, 279–288 10.1016/j.neuron.2005.09.024 16242408

[9] Yelshanskaya M. V., Singh A. K., Sampson J. M., Narangoda C., Kurnikova M., and Sobolevsky A. I. (2016) Structural bases of noncompetitive inhibition of AMPA-subtype ionotropic glutamate receptors by antiepileptic drugs. Neuron 91, 1305–1315 10.1016/j.neuron.2016.08.012 27618672 PMC5033713

[10] Maher M. P., Wu N., Ravula S., Ameriks M. K., Savall B. M., Liu C., Lord B., Wyatt R. M., Matta J. A., Dugovic C., Yun S., Ver Donck L., Steckler T., Wickenden A. D., Carruthers N. I., et al (2016) Discovery and characterization of AMPA receptor modulators selective for TARP-γ8. J. Pharmacol. Exp. Ther. 357, 394–414 10.1124/jpet.115.231712 26989142

[11] Kato A. S., Burris K. D., Gardinier K. M., Gernert D. L., Porter W. J., Reel J., Ding C., Tu Y., Schober D. A., Lee M. R., Heinz B. A., Fitch T. E., Gleason S. D., Catlow J. T., Yu H., et al (2016) Forebrain-selective AMPA-receptor antagonism guided by TARP γ-8 as an antiepileptic mechanism. Nat. Med. 22, 1496–1501 10.1038/nm.4221 27820603

[12] Gardinier K. M., Gernert D. L., Porter W. J., Reel J. K., Ornstein P. L., Spinazze P., Stevens C. C., Hahn P., Hollinshead S. P., Mayhugh D., Schkeryantz J., Khilevich A., De Frutos O., Gleason S. D., Kato A. S., et al (2016) Discovery of the first α-amino-3-hydroxy-5-methyl-4-isoxazolepropionic acid (AMPA) receptor antagonist dependent upon transmembrane AMPA receptor regulatory protein (TARP) γ-8. J. Med. Chem. 59, 4753–4768 10.1021/acs.jmedchem.6b00125 27067148

[13] Savall B. M., Wu D., Swanson D. M., Seierstad M., Wu N., Vives Martinez J., García Olmos B., Lord B., Coe K., Koudriakova T., Lovenberg T. W., Carruthers N. I., Maher M. P., and Ameriks M. K. (2019) Discovery of imidazo[1,2- *a*]pyrazines and pyrazolo[1,5- c]pyrimidines as TARP γ-8 selective AMPAR negative modulators. ACS Med. Chem. Lett. 10, 267–272 10.1021/acsmedchemlett.8b00599 30891124 PMC6421542

[14] Azumaya C. M., Days E. L., Vinson P. N., Stauffer S., Sulikowski G., Weaver C. D., and Nakagawa T. (2017) Screening for AMPA receptor auxiliary subunit specific modulators. PLoS ONE. 12, e0174742 10.1371/journal.pone.0174742 28358902 PMC5373622

[15] Herguedas B., Krieger J., and Greger I. H. (2013) Receptor heteromeric assembly-how it works and why it matters: The case of ionotropic glutamate receptors. in Prog. Mol. Biol. Transl. Sci., 117, pp. 361–386, Elsevier 23663975 10.1016/B978-0-12-386931-9.00013-1

[16] Schwenk J., Harmel N., Brechet A., Zolles G., Berkefeld H., Müller C. S., Bildl W., Baehrens D., Hüber B., Kulik A., Klöcker N., Schulte U., and Fakler B. (2012) High-resolution proteomics unravel architecture and molecular diversity of native AMPA receptor complexes. Neuron 74, 621–633 10.1016/j.neuron.2012.03.034 22632720

[17] Jackson A. C., and Nicoll R. A. (2011) The expanding social network of ionotropic glutamate receptors: TARPs and other transmembrane auxiliary subunits. Neuron 70, 178–199 10.1016/j.neuron.2011.04.007 21521608 PMC3119519

[18] Greger I. H., Watson J. F., and Cull-Candy S. G. (2017) Structural and functional architecture of AMPA-type glutamate receptors and their auxiliary proteins. Neuron 94, 713–730 10.1016/j.neuron.2017.04.009 28521126

[19] Yamazaki M., Fukaya M., Hashimoto K., Yamasaki M., Tsujita M., Itakura M., Abe M., Natsume R., Takahashi M., Kano M., Sakimura K., and Watanabe M. (2010) TARPs γ-2 and γ-7 are essential for AMPA receptor expression in the cerebellum. Eur. J. Neurosci. 31, 2204–2220 10.1111/j.1460-9568.2010.07254.x 20529126

[20] Chen L., Chetkovich D. M., Petralia R. S., Sweeney N. T., Kawasaki Y., Wenthold R. J., Bredt D. S., and Nicoll R. A. (2000) Stargazin regulates synaptic targeting of AMPA receptors by two distinct mechanisms. Nature. 408, 936–943 10.1038/35050030 11140673

[21] Straub C., and Tomita S. (2012) The regulation of glutamate receptor trafficking and function by TARPs and other transmembrane auxiliary subunits. Curr. Opin. Neurobiol. 22, 488–495 10.1016/j.conb.2011.09.005 21993243 PMC3265624

[22] Kato A. S., Gill M. B., Yu H., Nisenbaum E. S., and Bredt D. S. (2010) TARPs differentially decorate AMPA receptors to specify neuropharmacology. Trends Neurosci. 33, 241–248 10.1016/j.tins.2010.02.004 20219255

[23] Letts V. A., Felix R., Biddlecome G. H., Arikkath J., Mahaffey C. L., Valenzuela A., Bartlett F. S., Mori Y., Campbell K. P., and Frankel W. N. (1998) The mouse stargazer gene encodes a neuronal Ca2+-channel γ subunit. Nat. Genet. 19, 340–347 10.1038/1228 9697694

[24] Rouach N., Byrd K., Petralia R. S., Elias G. M., Adesnik H., Tomita S., Karimzadegan S., Kealey C., Bredt D. S., and Nicoll R. A. (2005) TARP γ-8 controls hippocampal AMPA receptor number, distribution and synaptic plasticity. Nat. Neurosci. 8, 1525–1533 10.1038/nn1551 16222232

[25] Kato A. S., Gill M. B., Ho M. T., Yu H., Tu Y., Siuda E. R., Wang H., Qian Y. W., Nisenbaum E. S., Tomita S., and Bredt D. S. (2010) Hippocampal AMPA receptor gating controlled by both tarp and cornichon proteins. Neuron 68, 1082–1096 10.1016/j.neuron.2010.11.026 21172611 PMC3034222

[26] Schwenk J., Harmel N., Zolles G., Bildl W., Kulik A., Heimrich B., Chisaka O., Jonas P., Schulte U., Fakler B., and Klöcker N. (2009) Functional proteomics identify cornichon proteins as auxiliary subunits of AMPA receptors. Science 323, 1313–1319 10.1126/science.1167852 19265014

[27] Schwenk J., Baehrens D., Haupt A., Bildl W., Boudkkazi S., Roeper J., Fakler B., and Schulte U. (2014) Regional diversity and developmental dynamics of the AMPA-receptor proteome in the mammalian brain. Neuron 84, 41–54 10.1016/j.neuron.2014.08.044 25242221

[28] Zhao Y., Chen S., Yoshioka C., Baconguis I., and Gouaux E. (2016) Architecture of fully occupied GluA2 AMPA receptor-TARP complex elucidated by cryo-EM. Nature. 536, 108–111 10.1038/nature18961 27368053 PMC4998972

[29] Twomey E. C., Yelshanskaya M. V., Grassucci R. A., Frank J., and Sobolevsky A. I. (2016) Elucidation of AMPA receptor-stargazin complexes by cryo-electron microscopy. Science 353, 83–86 10.1126/science.aaf8411 27365450 PMC5125255

[30] Ben-Yaacov A., Gillor M., Haham T., Parsai A., Qneibi M., and Stern-Bach Y. (2017) Molecular mechanism of AMPA receptor modulation by TARP/Stargazin. Neuron 93, 1126–1137.e4 10.1016/j.neuron.2017.01.032 28238551

[31] Maher M. P., Matta J. A., Gu S., Seierstad M., and Bredt D. S. (2017) Getting a handle on neuropharmacology by targeting receptor-associated proteins. Neuron 96, 989–1001 10.1016/j.neuron.2017.10.001 29216460

[32] Kato A. S., and Witkin J. M. (2018) Protein complexes as psychiatric and neurological drug targets. Biochem. Pharmacol. 151, 263–281 10.1016/j.bcp.2018.01.018 29330067

[33] Knopp K. L., Simmons R. M. A., Guo W., Adams B. L., Gardinier K. M., Gernert D. L., Ornstein P. L., Porter W., Reel J., DIng C., Wang H., Qian Y., Burris K. D., Need A., Barth V., et al (2019) Modulation of TARP γ8-containing AMPA receptors as a novel therapeutic approach for chronic pain. J. Pharmacol. Exp. Ther. 369, 345–363 10.1124/jpet.118.250126 30910921

[34] Herguedas B., Watson J. F., Ho H., Cais O., García-Nafría J., and Greger I. H. (2019) Architecture of the heteromeric GluA1/2 AMPA receptor in complex with the auxiliary subunit TARP γ8. Science 364, eaav9011 10.1126/science.aav9011 30872532 PMC6513756

[35] Ravula S., Savall B. M., Wu N., Lord B., Coe K., Wang K., Seierstad M., Swanson D. M., Ziff J., Nguyen M., Leung P., Rynberg R., La D., Pippel D. J., Koudriakova T., et al (2018) Lead optimization of 5-aryl benzimidazolone- and oxindole-based AMPA receptor modulators selective for TARP γ-8. ACS Med. Chem. Lett. 9, 821–826 10.1021/acsmedchemlett.8b00215 30128074 PMC6088354

[36] Lee M. R., Gardinier K. M., Gernert D. L., Schober D. A., Wright R. A., Wang H., Qian Y., Witkin J. M., Nisenbaum E. S., and Kato A. S. (2017) Structural determinants of the γ-8 TARP dependent AMPA receptor antagonist. ACS Chem. Neurosci. 8, 2631–2647 10.1021/acschemneuro.7b00186 28825787

[37] Gottlieb H. E., Kotlyar V., and Nudelman A. (1997) NMR chemical shifts of common laboratory solvents as trace impurities. J. Org. Chem. 62, 7512–7515 10.1021/jo971176v 11671879

[38] Šali A., and Blundell T. L. (1993) Comparative protein modelling by satisfaction of spatial restraints. J. Mol. Biol. 234, 779–815 10.1006/jmbi.1993.1626 8254673

[39] Shen M.-Y., and Sali A. (2006) Statistical potential for assessment and prediction of protein structures. Protein Sci. 15, 2507–2524 10.1110/ps.062416606 17075131 PMC2242414

[40] Trott O., and Olson A. J. (2010) AutoDock Vina: improving the speed and accuracy of docking with a new scoring function, efficient optimization, and multithreading. J. Comput. Chem. 31, 455–461 10.1002/jcc.21334 19499576 PMC3041641

[41] Jo S., Kim T., Iyer V. G., and Im W. (2008) CHARMM-GUI: a web-based graphical user interface for CHARMM. J. Comput. Chem. 29, 1859–1865 10.1002/jcc.20945 18351591

[42] Halgren T. A., Murphy R. B., Friesner R. A., Beard H. S., Frye L. L., Pollard W. T., and Banks J. L. (2004) Glide: a new approach for rapid, accurate docking and scoring. 2. enrichment factors in database screening. J. Med. Chem. 47, 1750–1759 10.1021/jm030644s 15027866

[43] Sherman W., Day T., Jacobson M. P., Friesner R. A., and Farid R. (2006) Novel procedure for modeling ligand/receptor induced fit effects. J. Med. Chem. 49, 534–553 10.1021/jm050540c 16420040

[44] Vanommeslaeghe K., Hatcher E., Acharya C., Kundu S., Zhong S., Shim J., Darian E., Guvench O., Lopes P., Vorobyov I., and Mackerell A. D. (2010) CHARMM general force field: a force field for drug-like molecules compatible with the CHARMM all-atom additive biological force fields. J. Comput. Chem. 31, 671–690 10.1002/jcc.21367 19575467 PMC2888302

[45] Shi Y., Lu W., Milstein A. D., and Nicoll R. A. (2009) The stoichiometry of AMPA receptors and TARPs varies by neuronal cell type. Neuron 62, 633–640 10.1016/j.neuron.2009.05.016 19524523 PMC3119531

[46] Cho C. H., St-Gelais F., Zhang W., Tomita S., and Howe J. R. (2007) Two families of TARP isoforms that have distinct effects on the kinetic properties of AMPA receptors and synaptic currents. Neuron 55, 890–904 10.1016/j.neuron.2007.08.024 17880893

[47] Milstein A. D., Zhou W., Karimzadegan S., Bredt D. S., and Nicoll R. A. (2007) TARP subtypes differentially and dose-dependently control synaptic AMPA receptor gating. Neuron 55, 905–918 10.1016/j.neuron.2007.08.022 17880894 PMC3167227

[48] Carbone A. L., and Plested A. J. R. (2016) Superactivation of AMPA receptors by auxiliary proteins. Nat. Commun. 7, 10178 10.1038/ncomms10178 26744192 PMC4729862

[49] Gill M. B., Kato A. S., Roberts M. F., Yu H., Wang H., Tomita S., and Bredt D. S. (2011) Cornichon-2 modulates AMPA receptor-transmembrane AMPA receptor regulatory protein assembly to dictate gating and pharmacology. J. Neurosci. 31, 6928–6938 10.1523/JNEUROSCI.6271-10.2011 21543622 PMC4562416

[50] Hawken N. M., Zaika E. I., and Nakagawa T. (2017) Engineering defined membrane-embedded elements of AMPA receptor induces opposing gating modulation by cornichon 3 and stargazin. J. Physiol. 595, 6517–6539 10.1113/JP274897 28815591 PMC5638889

[51] Carrillo E., Shaikh S. A., Berka V., Durham R. J., Litwin D. B., Lee G., MacLean D. M., Nowak L. M., and Jayaraman V. (2020) Mechanism of modulation of AMPA receptors by TARP-γ8. J. Gen. Physiol. 10.1085/jgp.201912451 31748249 PMC7034100

[52] Altschul S. F., Madden T. L., Schäffer A. A., Zhang J., Zhang Z., Miller W., and Lipman D. J. (1997) Gapped BLAST and PSI-BLAST: A new generation of protein database search programs. Nucleic Acids Res. 25, 3389–3402 10.1093/nar/25.17.3389 9254694 PMC146917

[53] Šali A., and Overington J. P. (1994) Derivation of rules for comparative protein modeling from a database of protein structure alignments. Protein Sci. 3, 1582–1596 10.1002/pro.5560030923 7833817 PMC2142932

[54] Fiser A., and Sali A. (2003) ModLoop: automated modeling of loops in protein structures. Bioinformatics 19, 2500–2501 10.1093/bioinformatics/btg362 14668246

[55] Lee J., Cheng X., Swails J. M., Yeom M. S., Eastman P. K., Lemkul J. A., Wei S., Buckner J., Jeong J. C., Qi Y., Jo S., Pande V. S., Case D. A., Brooks C. L., MacKerell A. D., et al (2016) CHARMM-GUI input generator for NAMD, GROMACS, AMBER, OpenMM, and CHARMM/OpenMM simulations using the CHARMM36 additive force field. J. Chem. Theory Comput. 12, 405–413 10.1021/acs.jctc.5b00935 26631602 PMC4712441

[56] Lee J., Patel D. S., Ståhle J., Park S. J., Kern N. R., Kim S., Lee J., Cheng X., Valvano M. A., Holst O., Knirel Y. A., Qi Y., Jo S., Klauda J. B., Widmalm G., et al (2019) CHARMM-GUI membrane builder for complex biological membrane simulations with glycolipids and lipoglycans. J. Chem. Theory Comput. 15, 775–786 10.1021/acs.jctc.8b01066 30525595

[57] Corradi V., Mendez-Villuendas E., Ingólfsson H. I., Gu R. X., Siuda I., Melo M. N., Moussatova A., Degagné L. J., Sejdiu B. I., Singh G., Wassenaar T. A., Delgado Magnero K., Marrink S. J., and Tieleman D. P. (2018) Lipid-protein interactions are unique fingerprints for membrane proteins. ACS Cent. Sci. 4, 709–717 10.1021/acscentsci.8b00143 29974066 PMC6028153

[58] Yu W., He X., Vanommeslaeghe K., and MacKerell A. D. (2012) Extension of the CHARMM general force field to sulfonyl-containing compounds and its utility in biomolecular simulations. J. Comput. Chem. 33, 2451–2468 10.1002/jcc.23067 [] 22821581 PMC3477297

[59] Jorgensen W. L., Chandrasekhar J., Madura J. D., Impey R. W., and Klein M. L. (1983) Comparison of simple potential functions for simulating liquid water. J. Chem. Phys. 79, 926–935 10.1063/1.445869

[60] Evans D. J., and Holian B. L. (1985) The Nose–Hoover thermostat. J. Chem. Phys. 83, 4069–4074 10.1063/1.449071

[61] Parrinello M., and Rahman A. (1981) Polymorphic transitions in single crystals: a new molecular dynamics method. J. Appl. Phys. 52, 7182–7190 10.1063/1.328693

[62] Huang J., Rauscher S., Nawrocki G., Ran T., Feig M., De Groot B. L., Grubmüller H., and MacKerell A. D. (2017) CHARMM36m: an improved force field for folded and intrinsically disordered proteins. Nat. Methods. 14, 71–73 10.1038/nmeth.4067 27819658 PMC5199616

[63] Berendsen H. J. C., van der Spoel D., and van Drunen R. (1995) GROMACS: a message-passing parallel molecular dynamics implementation. Comput. Phys. Commun. 91, 43–56 10.1016/0010-4655(95)00042-E

[64] Abraham M. J., Murtola T., Schulz R., Páll S., Smith J. C., Hess B., and Lindah E. (2015) Gromacs: High performance molecular simulations through multi-level parallelism from laptops to supercomputers. SoftwareX 1-2, 19–25 10.1016/j.softx.2015.06.001

[65] Schrödinger L. L. C. (2019) Schrödinger Release 2019-3, LigPrep, New York

[66] Jacobson M. P., Friesner R. A., Xiang Z., and Honig B. (2002) On the role of the crystal environment in determining protein side-chain conformations. J. Mol. Biol. 320, 597–608 10.1016/S0022-2836(02)00470-9 12096912

[67] García-Nafría J., Watson J. F., and Greger I. H. (2016) IVA cloning: a single-tube universal cloning system exploiting bacterial *i*n *v*ivo *a*ssembly. Sci. Rep. 6, 27459 27264908 10.1038/srep27459 PMC4893743

[68] Jeffrey G. A. (1997) An Introduction to Hydrogen Bonding, Oxford University Press, New York and Oxford

