# Supplementary material for: Characterizing the binding and function of TARP γ8-selective AMPA receptor modulators
Source: J Biol Chem. 2020 Aug 3;295(43):14565–77. doi: 10.1074/jbc.RA120.014135 (PMC7586208; doi:10.1074/jbc.RA120.014135)
Supplement: Supporting Information [file supp_295_43_14565__index.html]

Characterising the binding and function of TARP γ8-selective AMPA receptor modulators — Binding mode of TARP ᵯE;8 selective AMPA receptor modulators — Characterizing the binding and function of TARP γ8-selective AMPA receptor modulators — Binding mode of TARP γ8 selective AMPA receptor modulators — Supporting Information 

# Characterizing the binding and function of TARP γ8-selective AMPA receptor modulators

## Supporting Information

- Supporting Information (to be published online) - supp info
