# Supplementary material for: Characterizing the binding and function of TARP γ8-selective AMPA receptor modulators
Source: J Biol Chem. 2020 Aug 3;295(43):14565–77. doi: 10.1074/jbc.RA120.014135 (PMC7586208; doi:10.1074/jbc.RA120.014135)
Supplement: Supporting Information [file supp_RA120.014135_160516_2_supp_573716_qssgs5.docx]

**Supporting Information for**

**Characterising the binding and function of TARP 𝛾γ8-selective AMPA receptor modulators**

Jan-Niklas Dohrke, Jake F. Watson, Kristian Birchall and Ingo H. Greger

Correspondence to: ig@mrc-lmb.cam.ac.uk

This PDF file includes:

**Supporting Figures S1-6**

**Supporting Discussion**

Previous work on identifying the pose of LY-481 on a claudin-19 homology model of TARP 𝛾8 (Lee *et al.* 2017) suggested a pose, which we aimed to mimick in one simulation (**Fig.** **S5A1**). The earlier study did not predict binding via the oxindole group but still saw close alignment of the ligand with its pocket. To allow better comparison with the earlier work we also placed three water molecules into the binding pocket but could not replicate the previously reported binding mode with our TARP 𝛾8 model. Instead, we observed H-bonding of the primary hydroxyl group of the ligand’s variable region to the N172 side chain via water molecules (**Fig. S5B)**. The substructure in the pocket seems not to be very sensitive to the pIC_50_ value by large substitutions in SAR studies (**Fig. S1 B** and **C**).


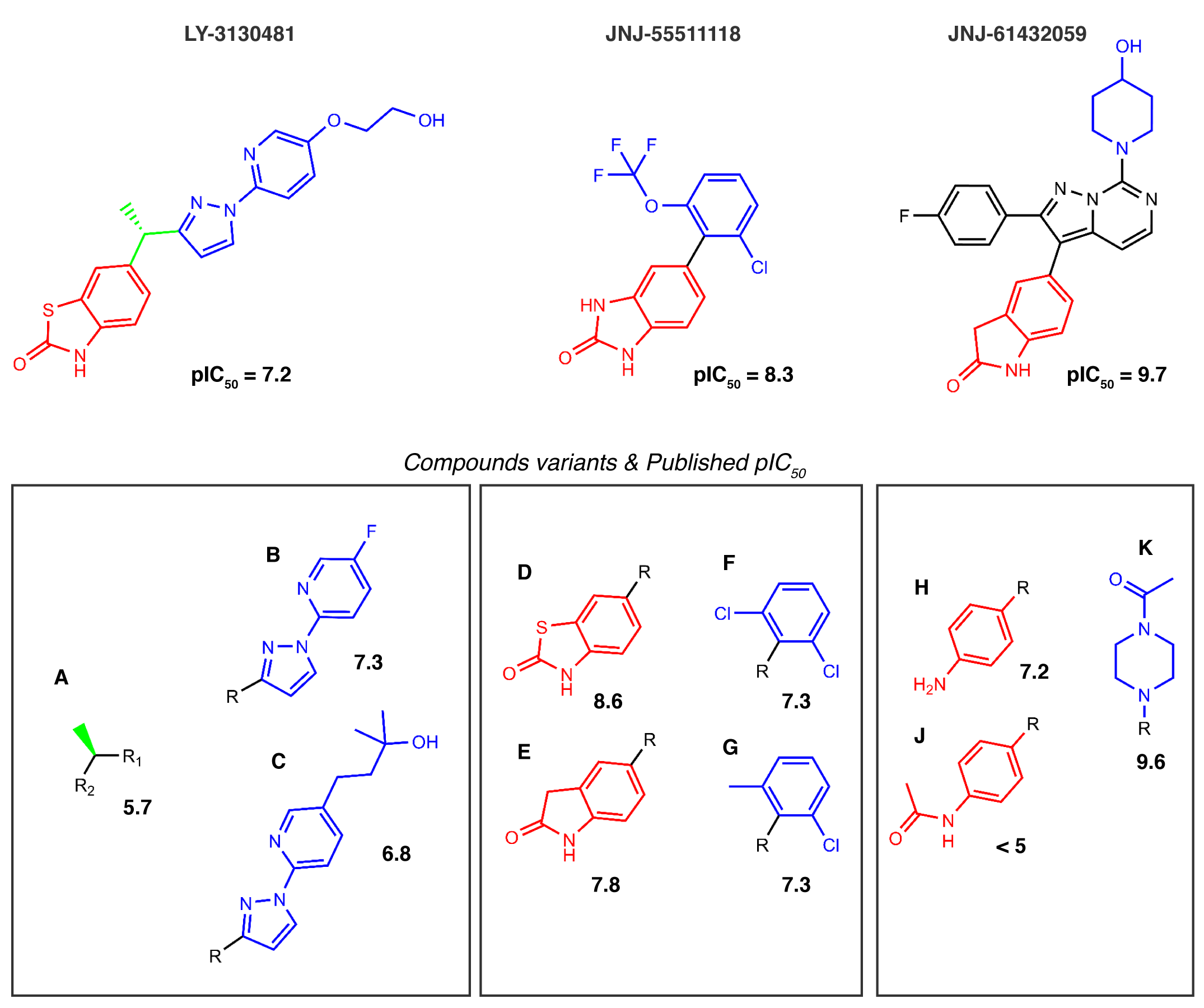


**Figure S1: Structure-activity relationship of TARP γ8-selective ligands.** Three TARP γ8-selective ligands and their reported pIC_50_ (from IC_50_ values in M) values. Variants of these ligands tested during lead optimization, and their corresponding pIC_50_ values are depicted below each ligand (LY-481: **A-C**, JNJ-118: **D-G**, JNJ-059: **H-K**). The oxindole group (red), variable region (blue) and chiral linker of LY-3130481 (green) are coloured for visualisation. Data from Gardinier *et al*., 2016 (LY-3130481), Ravula *et al*., 2018 (JNJ-55511118), and Savall *et al*., 2018 (JNJ-61432059). The rest (R) in the bottom row is the part of the corresponding top row structure, which is differently colored. Note: **H** and **J** differ in R from lead compound JNJ-61432059, and therefore for direct comparison should be considered in comparison to compound 11 of the original study.


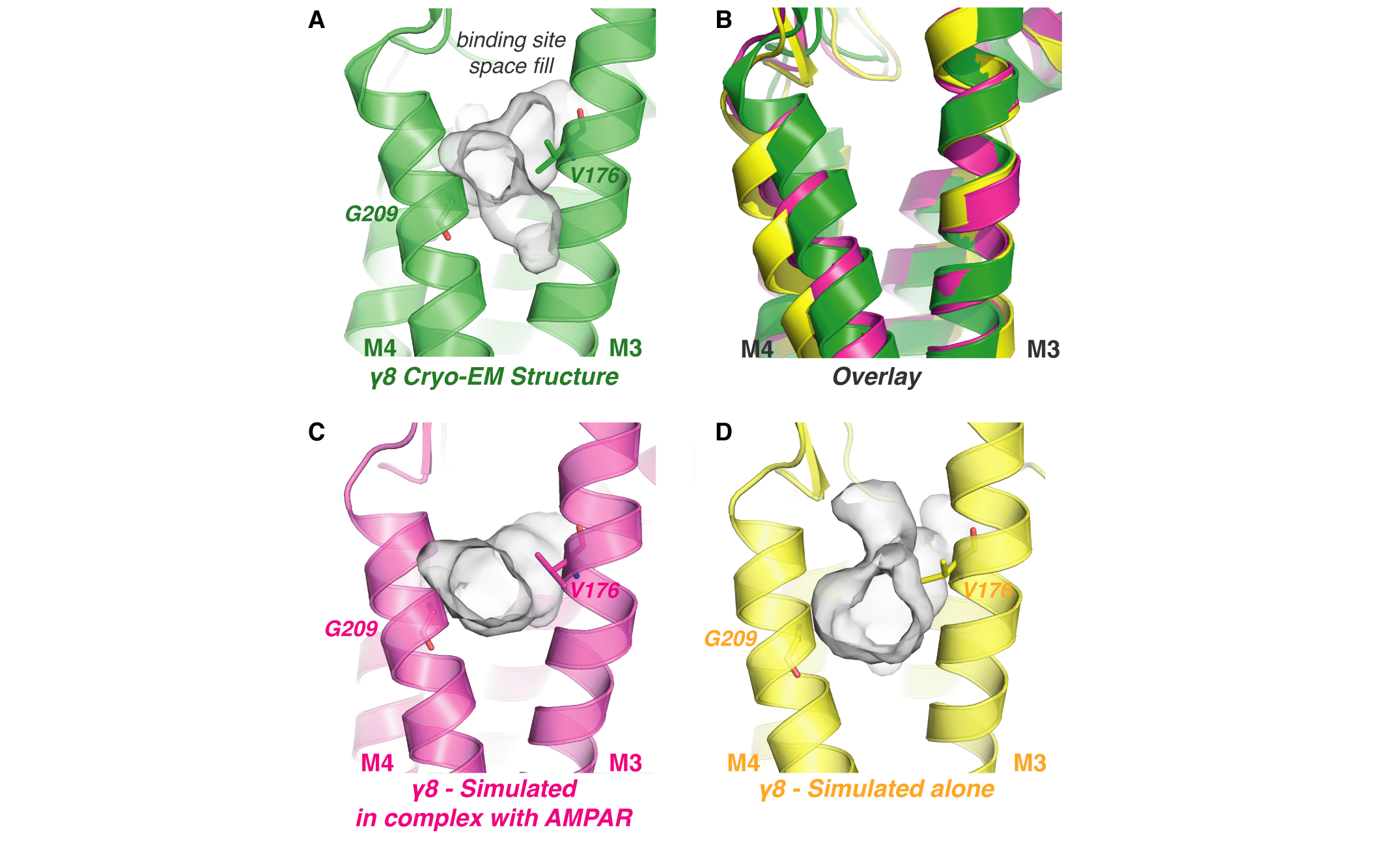


**Figure S2: Comparison of ligand-binding pocket size from structural data and MD simulations.** The ligand-binding pocket, between the M3 and M4 helices of TARP γ8, measured between the selectivity residues V176 and G209, can widen in MD simulations, from the published cryo-EM structural data PDB:6QKC (Herguedas *et al.* 2019).


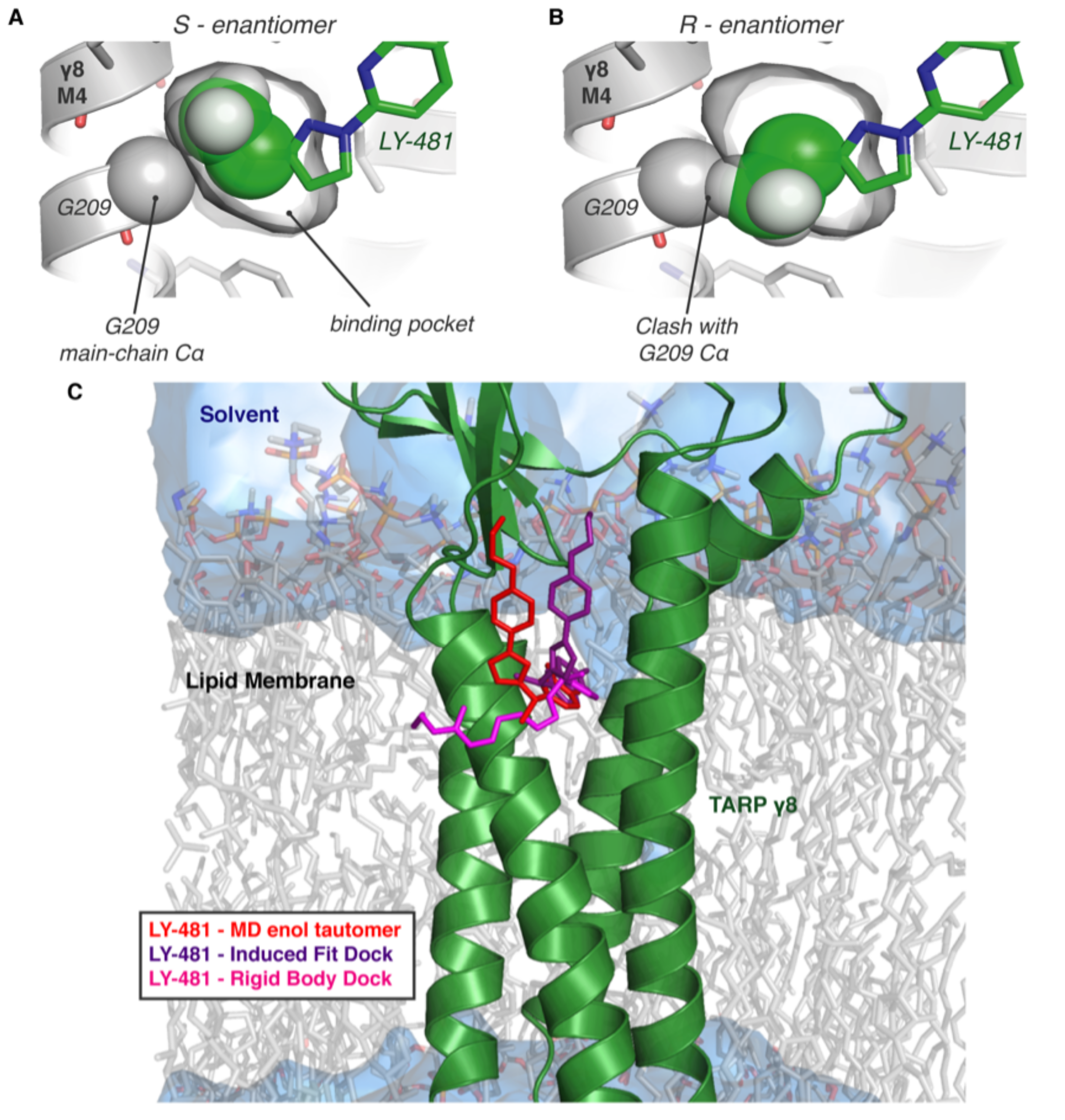


**Figure S3: LY-3130481 stereochemistry influences potential binding modes.** The R-enantiomer of LY-3130481 is less active than the S-enantiomer. The pose obtained from induced fit docking (**A**) demonstrate that the R-enantiomer (**B**) would clash with the surface of the binding pocket. **C** View of LY-481 poses in context of the lipid membrane (grey sticks) and aqueous regions (blue volumes). Three binding modes of LY-481 with TARP γ8 (green) are depicted, showing the ‘upright’ conformation of the ligand. Final poses from MD simulations (red), and Induced fit docking (purple) show association of LY-481 with the upper regions of TARP γ8 TM3 and TM4. Rigid Body Docking (pink) does not show this arrangement.


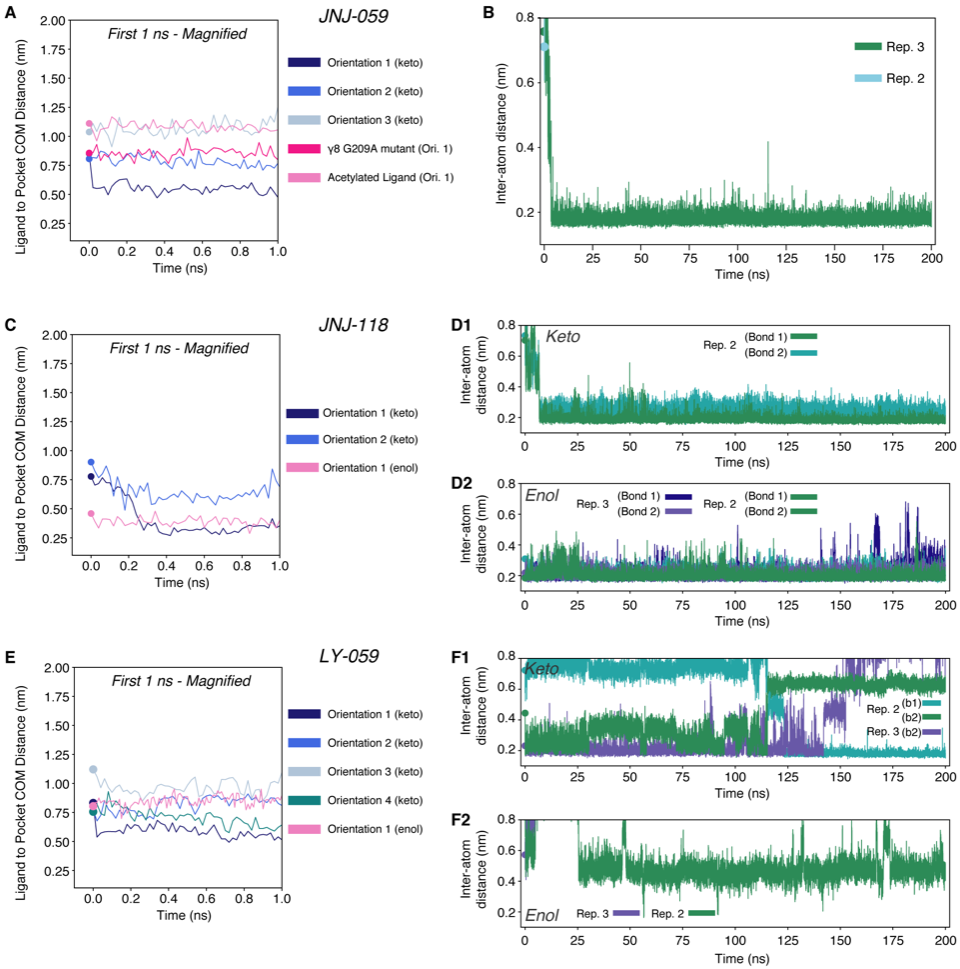


**Figure S4: Additional MD simulation results.** The initial behavior of the ligand, up to 1 ns in the simulation, is shown (**A**, **C** and **E**) from the corresponding simulation (**Fig. 4**, **5** and **6**). On the left, replicas are reported. Every replica was set up with orientation 1 of the corresponding figure as initial starting configuration: For **B** orientation 1 from **Fig. 4A**, for **D1/D2** from **Fig. 5A** (keto and enol respectively) and for **F1/F2** from **Fig. 6A** (keto and enol respectively). The replicas were equilibrated independently.


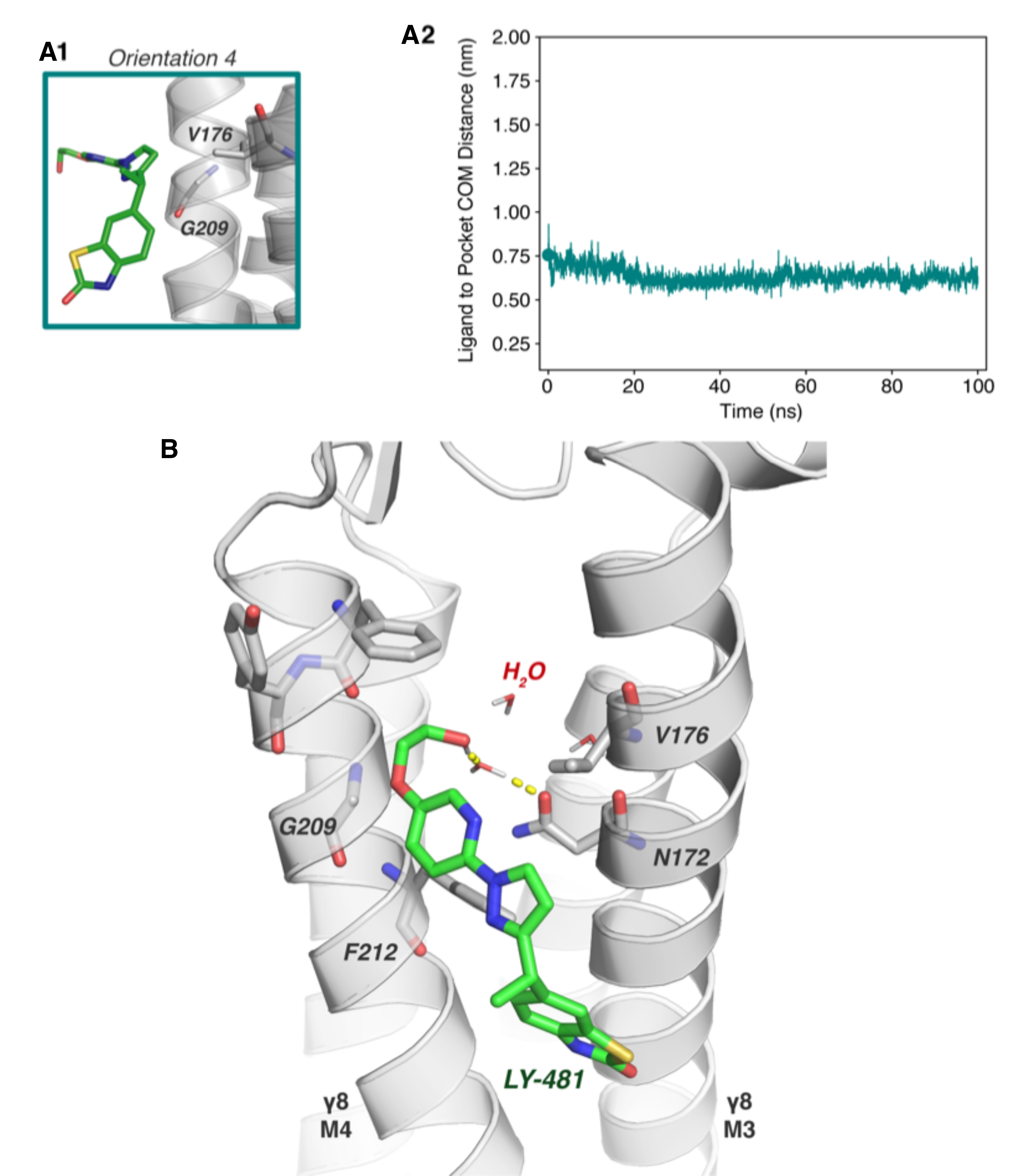


**Figure S5: Alternative binding of LY-3130481. A1** Orientation 4 mimics a binding mode described for LY-481 based in previous work (Lee *et al.* 2017). **A2** LY-481 finds a position close to the pockets center of mass (COM). **B** The final frame of orientation 4 simulation depicts a binding mode occurring via water molecules (sticks shown) in the pocket.


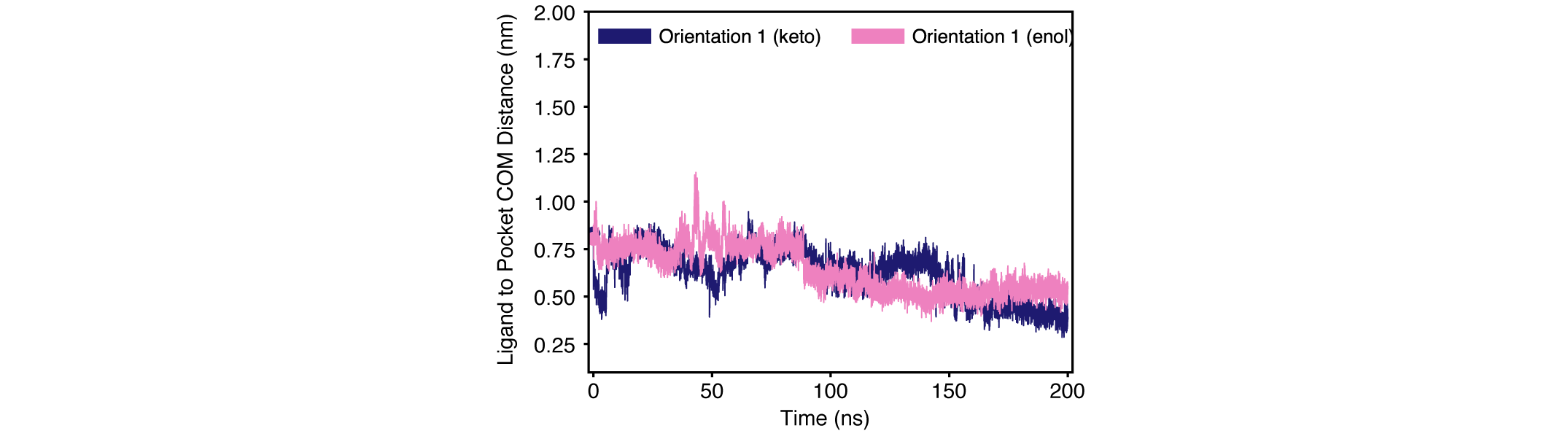


**Figure S6: Complete MD trajectory of LY-3130481 tautomers.** 200 ns simulation of both LY-481 tautomers in orientation 1 from **Fig. 6**. is shown. Note that ligand movement is limited after the binding event of the enol tautomer around 80 ns, while the keto tautomer maintains a fluctuating behaviour.

**Figure S7: Rise time of AMPAR currents are unaffected by modulatory compounds.** Rise time (for 20 to 80 % current increase) of AMPAR currents on glutamate application are unaffected by either compound (*LY,* Vehicle: 0.63 ± 0.02 ms, LY-481: 0.64 ± 0.03 ms, n = 20 cells, p=0.76; *JNJ,* Vehicle: 0.60 ± 0.04 ms, JNJ-118: 0.61 ± 0.04 ms, n = 12 cells, p=0.91; Wilcoxon tests).
